# The tumoural landscape of lymphocytes and immune pathways in immunotherapy-treated melanoma patients

**DOI:** 10.1038/s41435-026-00406-1

**Published:** 2026-07-15

**Authors:** Vinícius Gonçalves de Souza, Bruna Pereira Sorroche, Renan de Jesus Teixeira, Hendrigo Nunes, Ana Carolina Laus, Vinícius de Lima Vazquez, Daniele Moraes Losada, Lidia Maria Rebolho Batista Arantes

**Affiliations:** 1 Molecular Oncology Research Center, Barretos Cancer Hospital, São Paulo, Brazil; 2 Department of Pathology, Barretos Cancer Hospital, São Paulo, Brazil; 3 Melanoma, Sarcoma and Mesenchymal Tumors Surgery, Barretos Cancer Hospital, São Paulo, Brazil

**Keywords:** Immunotherapy, Cancer genetics, Gene expression, Tumour immunology

## Abstract

Tumor-infiltrating lymphocytes (TILs) shape melanoma behavior and response to immunotherapy, but the links between immune regulation, TIL patterning, and outcomes remain unclear. We retrospectively studied 32 primary melanoma samples from patients treated with anti–PD-1 therapy. Histopathologic TILs were classified as brisk or non-brisk, and primary tumors underwent targeted immune transcriptomic profiling (NanoString nCounter Human Immunology panel). External validation was performed with 96 TCGA-SKCM primary tumors. Brisk tumors displayed broad upregulation of immune transcripts, with enrichment of adhesion pathways (directed global significance scores – DGSS = 2.15) and MHC class II antigen presentation (DGSS = 2.128). Cross-cohort comparison identified 22 shared differentially expressed genes, with *ZAP70* remaining significant in both datasets. Brisk tumors also showed higher total TIL (*p* = 0.036), cytotoxic-cell (*p* = 0.0095), and Th1 (*p* = 0.027) scores. Response-associated genes differed by TIL pattern, and subgroup-specific gene scores predicted anti–PD-1 benefit in brisk (4-gene, Area under curve - AUC = 1.000) and non-brisk (3-gene, AUC = 0.852) tumors; the non-brisk score remained independently associated with response (*p* = 0.029). Higher scores were also associated with prolonged survival. Integrating histopathological TIL patterning with immune transcriptomics may refine prognostication and support immunotherapy stratification in melanoma.

## Introduction

Melanoma is an aggressive malignant neoplasm arising from melanocytes that primarily affects the skin. Even very small lesions of this type possess the potential to metastasize [1]. The most important prognostic factors for primary melanoma include Breslow thickness, the presence of histologically defined ulceration, and the mitotic rate (number of mitoses per mm²) [2].

The tumor microenvironment plays a central role in the initiation, progression, and therapeutic response of melanoma, constituting a dynamic network of cellular and acellular components that interact and collectively modulate disease behavior. Several studies have emphasized the importance of understanding the tumor microenvironment in predicting prognosis and treatment response [3–5].

In this context, the evaluation of immune infiltration has gained prominence. In melanoma, the term “tumor-infiltrating lymphocyte” (TIL) was introduced by Clark et al. in 1989 in a study that incorporated clinicopathologic variables to develop a prognostic model for stage I cutaneous melanoma. Clark defined TILs in melanoma as lymphocytes that infiltrate and destroy tumor cells during the vertical growth phase of the lesion, as assessed histologically [6]. TILs are evaluated histopathologically according to their density patterns, which are classified as brisk, non-brisk, or absent. In melanoma, the brisk pattern is characterized by diffuse lymphocytic infiltration throughout the entire tumor area or diffuse infiltration across the tumor base, the non-brisk pattern shows focal or patchy infiltration, and the absent pattern lacks any significant TILs [6, 7].

Currently, TIL patterns have been extensively studied for their prognostic value in survival and as predictive biomarkers for response to immune checkpoint inhibitor therapy in melanoma patients [8–11]. Accordingly, several studies have sought to elucidate the molecular pathways associated with TIL infiltration to better understand the role of these cells in tumor progression and patient prognosis [12, 13]. However, the link between immune pathway regulation, TIL infiltration, and clinical outcomes remains to be fully elucidated.

Therefore, this study aimed to evaluate the association between immune-related gene expression profiles, TIL patterns, and clinical outcomes in melanoma patients treated with immunotherapy.

## Material and methods

### Patients

This was a retrospective observational study including 32 patients diagnosed with melanoma at a tertiary cancer center in Brazil. All patients received anti–PD-1 therapy (nivolumab or pembrolizumab) in monotherapy, either as first-line treatment or in subsequent lines of palliative care. Immunotherapy was indicated when patients presented with advanced disease, defined as unresectable clinical stage III or IV. Patients had not received prior anti-CTLA-4 therapy before anti-PD-1 treatment. Eligibility criteria included the availability of formalin-fixed paraffin-embedded (FFPE) samples from the primary tumor (baseline samples, without any systemic treatment) containing at least 60% tumor content and evidence of inflammatory infiltrate on histopathological slides. From the original cohort of 35 patients [14], three cases were classified as absent TILs and were excluded from this study, as the analyses were designed to compare tumors with detectable lymphocytic infiltration according to brisk and non-brisk patterns. This study was conducted according to the declaration of Helsinki and Brazilian National Research Ethics Commission. The study protocol was approved by the Institutional Review Board (CAAE: 12855019.6.0000.5437). The requirement for informed consent was waived by the Institutional Review Board/Ethics Committee due to the retrospective nature of the study.

### Histopathological evaluation

Formalin-fixed, paraffin-embedded tissue sections were cut at 5 μm and stained with hematoxylin and eosin (H&E) using the HistoCore SPECTRA ST Stainer (Leica Biosystems) according to the manufacturer’s instructions and routine institutional protocols. Briefly, sections were deparaffinized, rehydrated through graded ethanol, stained with hematoxylin and eosin, dehydrated, and coverslipped for microscopic evaluation. Histopathological analysis was independently performed by two experienced pathologists using all the slides available for each case. Cases were assessed according to the recommendations of the College of American Pathologists [15]. Tumor-infiltrating lymphocytes (TILs), the primary endpoint of the study, were classified as brisk (diffuse infiltration across the tumor base or diffuse permeation) or non-brisk (focal infiltrates). Cases without detectable TILs (TILs absent category) were excluded. Additional pathological variables evaluated included histological subtype, Breslow thickness, Clark level, ulceration, mitotic rate, lymphatic and/or vascular invasion, neurotropism, microsatellitosis, and tumor regression.

### Clinical evaluation

Clinical data were obtained from medical records. Anti–PD-1 therapy was administered until clinically significant toxicity, disease progression, or physician decision. Treatment response was assessed every 12 weeks according to the iRECIST guidelines [16]. Patients were categorized as responders (complete response, partial response or stable disease for ≥6 months) and non-responders (disease progression). Clinical outcomes analyzed included response to immunotherapy, overall survival (OS), progression-free survival (PFS), and post-immunotherapy survival (PIS). OS was calculated from the date of diagnosis to the date of death from any cause. PFS was calculated from the date of immunotherapy initiation to the date of first documented disease progression, either local or regional. PIS was calculated from the date of immunotherapy initiation to the date of death from any cause.

### Gene expression profiling

For gene expression profiling, RNA from FFPE primary tumor was extracted with the RNeasy FFPE Mini Kit (Qiagen, Hilden, Germany). The NanoString nCounter Human Immunology v2 Panel was used on the nCounter MAX Analysis System (NanoString Technologies, Seattle, WA, USA), with 250 ng of RNA as input per sample. The panel included 594 targets and 15 internal reference genes. The complete gene list is presented in Supplementary Table 1A.

### Differential expression analysis

Raw counts were acquired using the nCounter MAX Digital Analyzer (555 fields of view) and processed with nSolver Analysis Software (v4.0; NanoString Technologies) with the Advanced Analysis module. Genes with < 20 counts across all samples were excluded. For differential expression analysis, the adaptive model-selection strategy (“Optimal method”) was applied. For each gene, the normalized counts were evaluated considering the background noise and true expression. Three models were considered, a mixture negative binomial model (for low expression probes), a simplified negative binomial model (for high expression probes) and log-linear model (for genes that fail in the previous two models). The selection of appropriate models of analysis was automatically performed in nSolver Analysis Software according to fabricant algorithm. Differential expression analysis was adjusted by Clark’s level and Breslow thickness. Significant results considered if *p* < 0.05 and Fold-change (FC) ≥ ± 1.5. The Benjamini–Hochberg (BH) test was used for multiple comparisons.

### Pathway analysis

Pathway-level signals were assessed using the Gene Set Analysis (GSA) and Pathway Scoring modules from the nSolver Advanced Analysis suite (NanoString Technologies). GSA module considered a t-statistic test for each gene and aggregates this evidence at the gene-set level to compute global significance scores. The directed global significance score also incorporates in the analysis the direction of change by using the sign of each t-statistic test. Pathway analysis was also evaluated at sample-level with pathway scoring module. The analysis was based on the first principal component (PC1) of the genes’ normalized expression with each set. The BH test was used for multiple comparisons. Genes included in each pathway were presented in Supplementary Table 1B.

### Cell type analysis

Immune cell abundances were estimated with the Cell Type Profiling module from the nSolver Advanced Analysis suite, which infers cell populations based on predefined cell type-specific marker genes annotated in the panel. Raw cell type abundance scores were evaluated considering the mean log2 normalized counts of cell type-specific marker genes. Relative abundances were calculated using raw scores and gene expression-based definition of TILs, defined as the average of the B-cell, T-cell, CD45, macrophage, and cytotoxic cell scores. Group comparisons were performed using two-sided Wilcoxon rank-sum tests. BH test was used for multiple comparisons.

### Analysis of clinical outcomes

Genes differentially expressed according to immunotherapy response were identified separately in the brisk and non-brisk cohorts using nSolver Advanced Analysis, as described above.

Gene scores were developed separately for the brisk and non-brisk groups. Candidate genes were first filtered using a threshold of *p* < 0.01. To reduce redundancy, highly correlated genes were pruned using pairwise Spearman correlation, with one gene removed from each pair showing an absolute correlation coefficient of ≥0.80. Remaining genes were then subjected to elastic net penalized logistic regression (α = 0.5) with bootstrap resampling (1000 iterations). Only genes with at least 70% of valid bootstrap iterations were selected. The score was calculated as the mean z-scored expression of genes associated with response minus the mean z-scored expression of genes associated with non-response. Both raw and standardized score values were generated for each sample. The predictive performance of the score for immunotherapy response was assessed by receiver operating characteristic (ROC) curve analysis.

Multivariable logistic regression analyses for immunotherapy response were performed using Firth penalized logistic regression. Each variable was first evaluated in an univariable logistic regression model, and variables with *p* < 0.10 were retained for multivariable analysis. Variables yielding unstable or non-estimable coefficients due to singularity, aliasing, or related collinearity issues were excluded from the final analysis.

The prognostic value of the immunotherapy-related genes and gene score was then evaluated separately in the brisk and non-brisk groups using univariable Cox proportional hazards regression.

### External validation using The Cancer Genome Atlas (TCGA)

Validation of differentially expressed genes was performed using the TCGA-SKCM cohort. Digital H&E slides from TCGA primary melanoma cases were accessed through the Genomic Data Commons (GDC) portal and reviewed by the same evaluators who classified the discovery cohort cases. Cases whose available slides were not of sufficient quality for reliable evaluation or with absent TILs were excluded. Raw transcriptomic counts (STAR – Counts) were downloaded and used for the analysis. Data were normalized and analyzed for differential expression using edgeR, with significance defined as *p* < 0.05 and FC ≥ ± 1.5. Volcano plots and Venn diagrams were generated to evaluate overlaps between NanoString-derived and TCGA-derived gene sets. The same score formulation was applied to TCGA cases classified as brisk or non-brisk.

### Statistical analysis

Statistical analyses were conducted using IBM SPSS Statistics v23.0 (Chicago, IL, USA) and RStudio v2025.05.1 (The R Foundation for Statistical Computing, Vienna, Austria). Associations between categorical variables were evaluated with chi-square test, whereas differences in continuous variables were assessed using the Mann–Whitney U test. Survival curves were estimated with the Kaplan–Meier method and compared with the log-rank test. Cox proportional hazards regression was used for univariable and multivariable survival analyses. BH correction was applied where indicated for multiple testing. A *p*-value < 0.05 was considered statistically significant.

## Results

### Most immune-related genes and pathways were overexpressed in TILs brisk

**Fig. 1:**
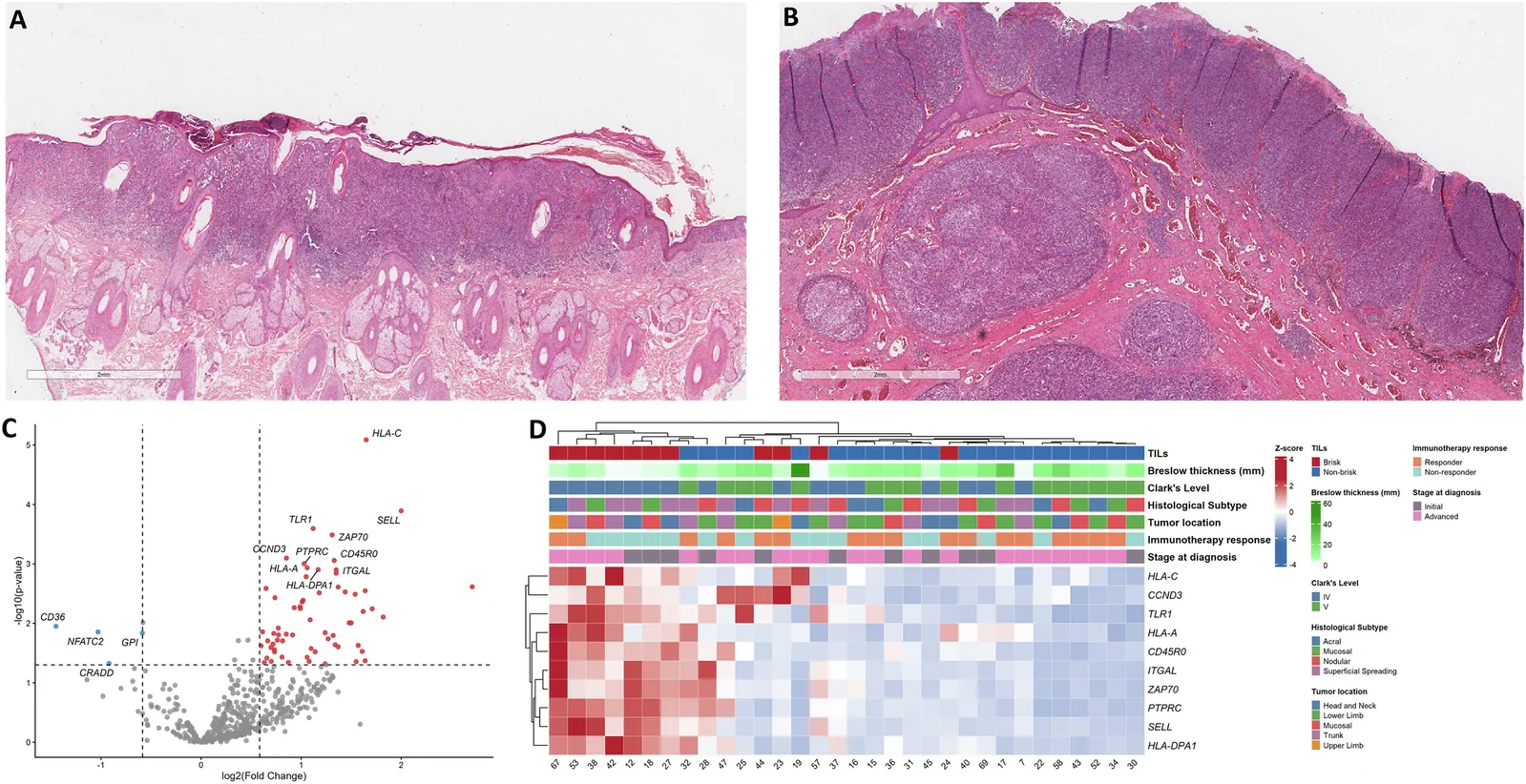
Histopathological classification and differential gene expression according to TIL pattern in primary melanoma. **A** Representative section of a melanoma with brisk Tumor Infiltrating Lymphocytes (TILs). **B** Representative section of a melanoma with non-brisk TILs. **C** Volcano plot showing differentially expressed genes between brisk and non-brisk tumors in the NanoString cohort. Red dots indicate genes upregulated in brisk tumors, whereas blue dots indicate genes upregulated in non-brisk tumors. **D** Heatmap showing the distribution of cases and the 10 most differentially expressed genes, together with selected clinical variables of interest.

Clinicopathological characterization of the Barretos Cancer Hospital cohort is presented in Table 1. Based on histopathological assessment of TILs, patients were classified as brisk (*n* = 11, Fig. 1A) or non-brisk (*n* = 21, Fig. 1B). Most brisk cases showed Clark’s level IV (81.8%) and lower Breslow thickness (4.2 mm), whereas most non-brisk cases showed Clark’s level V (65.0%) and higher Breslow thickness (8.0 mm) (*p* < 0.01 for both). Patient did not significantly differ regarding other clinicopathological characteristics (*p* > 0.05).

**Table 1:** Clinicopathological characterization of 32 melanoma patients included in Barretos Cancer Hospital cohort.

Characteristic	TILs pattern		*p*-value
	Brisk (*n* = 11)	Non-brisk (*n* = 21)	
Gender^a^			
Male	6 (54.5%)	13 (61.9%)	0.687
Female	5 (45.5%)	8 (38.1%)	
Age at diagnosis^b^	59.0 (45.0–77.0)	58.0 (25.0–79.0)	0.639
Tumor location^a^			
Lower limbs	2 (18.2%)	9 (42.9%)	0.21
Upper limbs	2 (18.2%)	0 (0.0%)	
Head and neck	3 (27.3%)	3 (14.3%)	
Trunk	2 (18.2%)	5 (23.8%)	
Mucosal	2 (18.2%)	4 (19.0%)	
Histological subtype^a^			
Superficial spreading	7 (63.9%)	5 (23.8%)	0.134
Nodular	1 (9.1%)	7 (33.3%)	
Acral	1 (9.1%)	5 (23.8%)	
Mucosal melanoma	2 (18.2%)	4 (19.0%)	
Breslow thickness (mm)^b^	4.2 (1.5–14.0)	8.0 (2.0–50.0)	**0.004**
Clark’s level^a^			
IV	9 (81.8%)	7 (35.0%)	**0.009**
V	2 (18.2%)	14 (65.0%)	
Ulceration^a^			
Absent	5 (45.5%)	6 (28.6%)	0.340
Present	6 (54.5%)	15 (71.4%)	
Mitotic rate (/mm²)^b^	9.0 (1.0–38.0)	10.0 (2.0–32.0)	0.845
Lymphatic and/or Vascular Invasion^a^			
Absent	8 (72.7%)	11 (52.4%)	0.266
Present	3 (27.3%)	10 (47.6%)	
Neurotropism^a^			
Absent	10 (90.9%)	20 (95.2%)	0.631
Present	1 (9.1%)	1 (4.8%)	
Microsatellites^a^			
Absent	11 (100.0%)	18 (85.7%)	0.188
Present	0 (0.0%)	3 (14.3%)	
Tumor regression^a^			
Absent	9 (81.8%)	19 (90.5%)	0.482
Present	2 (18.2%)	2 (9.5%)	
Clinicopathological staging at diagnosis^a^			
Early (stages I and II)	4 (36.4%)	7 (33.3%)	0.864
Advanced (stages III and IV)	7 (63.6%)	14 (66.7%)	
Immunotherapy treatment^a^			
Nivolumab	6 (54.5%)	15 (71.4%)	0.340
Pembrolizumab	5 (45.5%)	6 (28.6%)	
Line of immunotherapy treatment^a^			
First	10 (90.9%)	11 (52.4%)	0.091
Second	1 (9.1%)	9 (42.9%)	
Third or more	0 (0.0%)	1 (4.8%)	
Best immunotherapy response^a^			
Complete response	2 (18.2%)	1 (4.8%)	0.433
Partial response	1 (9.1%)	5 (23.8%)	
Stable disease	2 (18.2%)	6 (28.6%)	
Disease progression	6 (54.5%)	9 (42.9%)	
Immunotherapy response group^a^			
Responder	5 (45.5%)	12 (57.1%)	0.529
Non-responder	6 (54.5%)	9 (42.9%)	
Overall survival (months)^c^	30.1 (6.2–91.5)	39.5 (10.1–108.3)	0.897
Progression-free survival (months)^c^	3.1 (1.0–74.4)	9.9 (1.2–86.0)	0.917
Survival after immunotherapy (months)^c^	13.5 (1.5–74.4)	22.2 (3.4–86.0)	0.704

*TILs* Tumor-infiltrating lymphocytes.

^a^Value presented as absolute frequency (relative frequency). Statistical test: Chi-square.

^b^Value presented as median (minimum - maximum). Statistical test: Mann–Whitney.

^c^Value presented as median (minimum - maximum). Statistical test: Log-rank test.

Among the 594 genes evaluated, 68 were differentially expressed between TIL infiltration patterns (Fig. 1C). Of these, 64 genes were overexpressed in the TILs brisk group, while 4 were overexpressed in the non-brisk group. After BH test, *HLA-C*, *SELL*, *TLR1*, and *ZAP70* remained significantly overexpressed in the brisk group (adjusted *p* value < 0.05, Supplementary Table 1C). Complete differential expression results are provided in Supplementary Table 1C. The heatmap depicts the expression patterns of the top 10 genes across cases stratified by TIL status, distinguishing brisk from non-brisk infiltrates, together with associated clinicopathological characteristics (Fig. 1D).

### TCGA validation confirmed part of the brisk-associated transcriptional profile

Among TCGA-SKCM cases, only primary tumors were considered for validation. A total of 104 primary tumor cases were initially identified. One case was excluded due to unavailable RNA-seq data, five cases were excluded due to inadequate histopathological images for review, and two cases were excluded because they were classified as having absent TILs, resulting in a final set of 96 evaluable cases. Of these, 51 cases were categorized as brisk and 45 as non-brisk (Supplementary Table 1D).

**Fig. 2:**
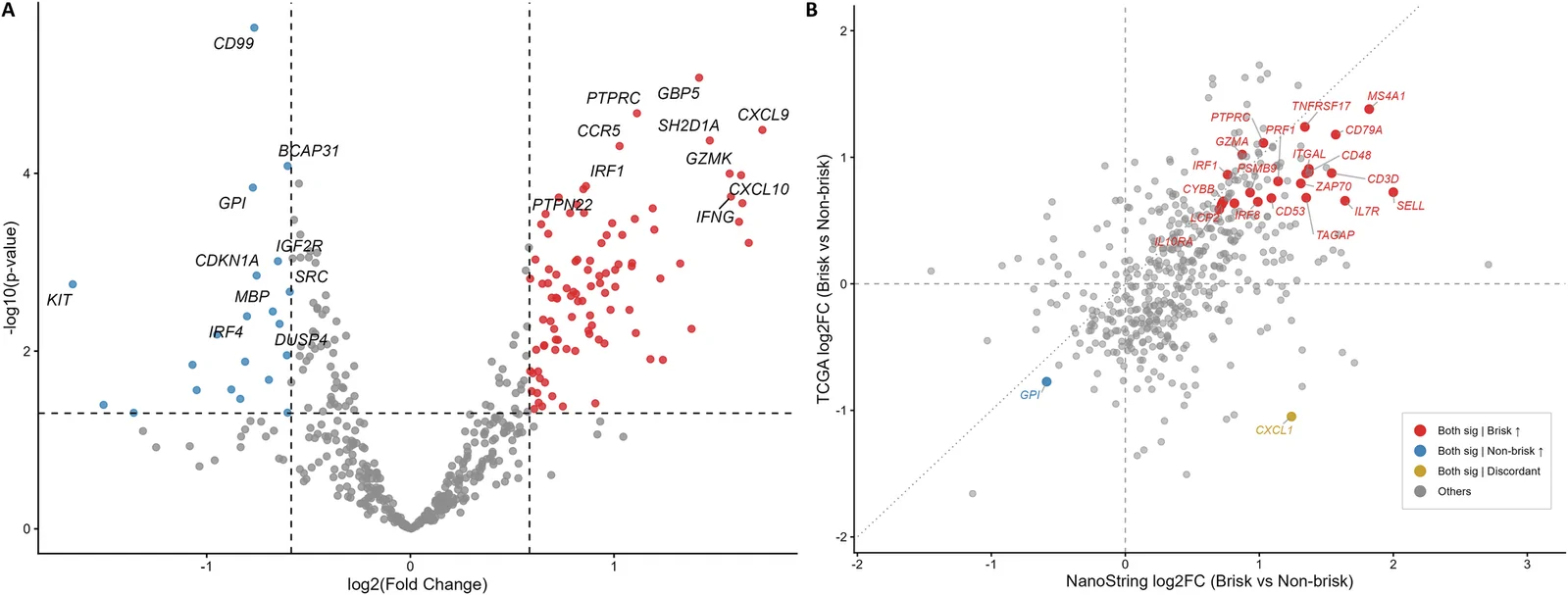
External validation of the brisk versus non-brisk transcriptional profile in the TCGA cohort. **A** Volcano plot showing differential gene expression between brisk and non-brisk tumors in the TCGA cohort. Red dots indicate genes upregulated in brisk tumors and blue dots indicate genes upregulated in non-brisk tumors. **B** Scatter plot comparing NanoString and TCGA fold-change values for the same genes. Genes significant in both datasets are highlighted according to direction of concordance: red, significant in both cohorts and higher in brisk tumors; blue, significant in both cohorts and higher in non-brisk tumors; yellow, significant in both cohorts but discordant in direction; gray, all remaining genes.

Transcriptomic profiling identified 80 genes overexpressed in brisk group and 30 genes overexpressed in non-brisk group (Fig. 2A, Supplementary Table 1E). Of these, 22 genes were also differentially expressed in the discovery (NanoString) cohort. Among them, *CXCL1* showed an inconsistent pattern: it was overexpressed in the brisk group in the NanoString cohort, but in the non-brisk group in the TCGA cohort (Fig. 2B). After correcting for multiple testing, only *ZAP70* remained significantly overexpressed in the brisk group in both cohorts (*p* < 0.05).

### Immune-related pathways were enriched in brisk TIL tumors

**Fig. 3:**
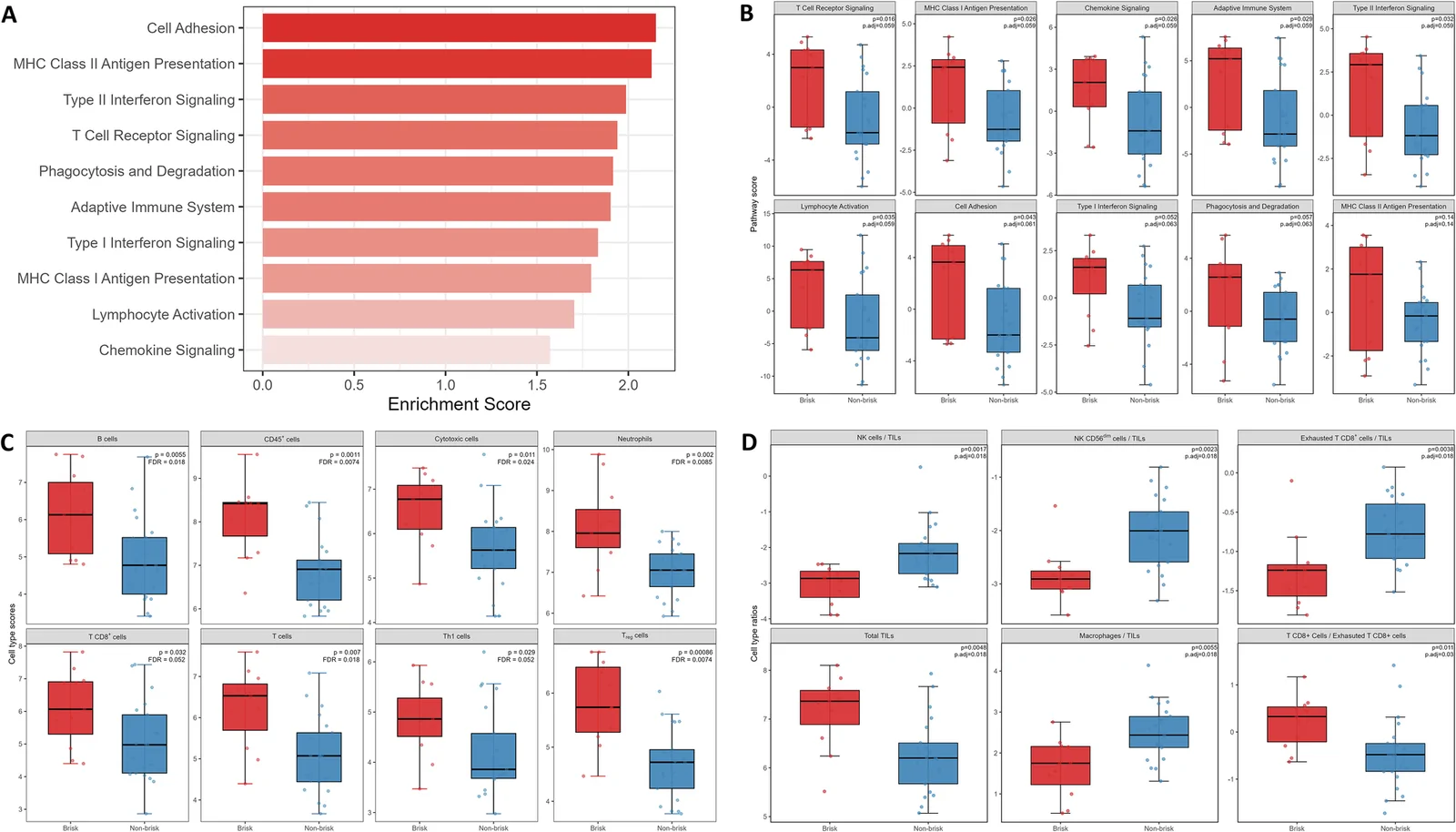
Pathway enrichment and immune cell composition associated with brisk TILs. **A** Top 10 enriched pathways identified in tumors with brisk TILs. Bars represent enrichment scores. **B** Boxplots showing sample-level pathway scores for the most relevant pathways, comparing brisk (red) and non-brisk (blue) tumors. **C** Boxplots of inferred immune cell type scores according to TIL pattern. **D** Boxplots of immune cell ratios and total TIL-related scores comparing brisk and non-brisk tumors. In boxplots, *n* = 11 for brisk group and *n* = 21 for non-brisk group.

The GSA module from the nSolver Advanced Analysis suite identified multiple pathways with high directed global significance scores (DGSS) in brisk group (Fig. 3A), particularly those involved in cell adhesion (DGSS = 2.15) and MHC Class II antigen presentation (DGSS = 2.128). Complete results of DGSS are presented in Supplementary Table 1F.

In the sample-level pathway analysis, although most brisk cases showed enrichment across several pathways, only T-cell receptor signaling, MHC class I antigen presentation, chemokine signaling, adaptive immune system, type II interferon signaling, and lymphocyte activation remained significant (*p* < 0.05) (Fig. 3B). None of these pathways remained significant after adjustment for multiple testing. Additional pathways are shown in Supplementary Fig. 1.

### Non-brisk tumors showed relatively higher proportions of exhausted T cells, macrophages, and NK cells

Cell-type scores derived from the NanoString immune panel showed that the brisk pattern was associated with higher levels of B cells, CD45⁺ cells, cytotoxic cells, neutrophils, T cells, and T_reg_ cells, and these differences remained significant after BH adjustment for multiple testing (adjusted *p* < 0.05; Fig. 3C). Higher Th1-cell and T CD8⁺-cell scores were also observed in brisk cases, but these associations did not remain significant after multiple-testing correction (nominal *p* < 0.05; adjusted *p* = 0.052 for both). No significant differences were observed for dendritic cells, exhausted T CD8⁺ cells, macrophages, NK CD56^dim^ cells, and NK cells (Supplementary Fig. 2A).

In addition, immune cell ratios indicated a higher proportion of total TILs and T CD8⁺ cells/exhausted T CD8⁺ cells in the brisk group, whereas the non-brisk group exhibited higher exhausted T CD8⁺cells/TILs, macrophages/TILs, NK CD56^dim^/TILs, and NK cells/TILs ratios. These associations remained significant after BH adjustment for multiple testing (adjusted *p* < 0.05; Fig. 3D). Other ratios did not differ significantly between the groups (Supplementary Fig. 2B).

### Predictive genes of immunotherapy response differ between TILs brisk and non-brisk patients

**Fig. 4:**
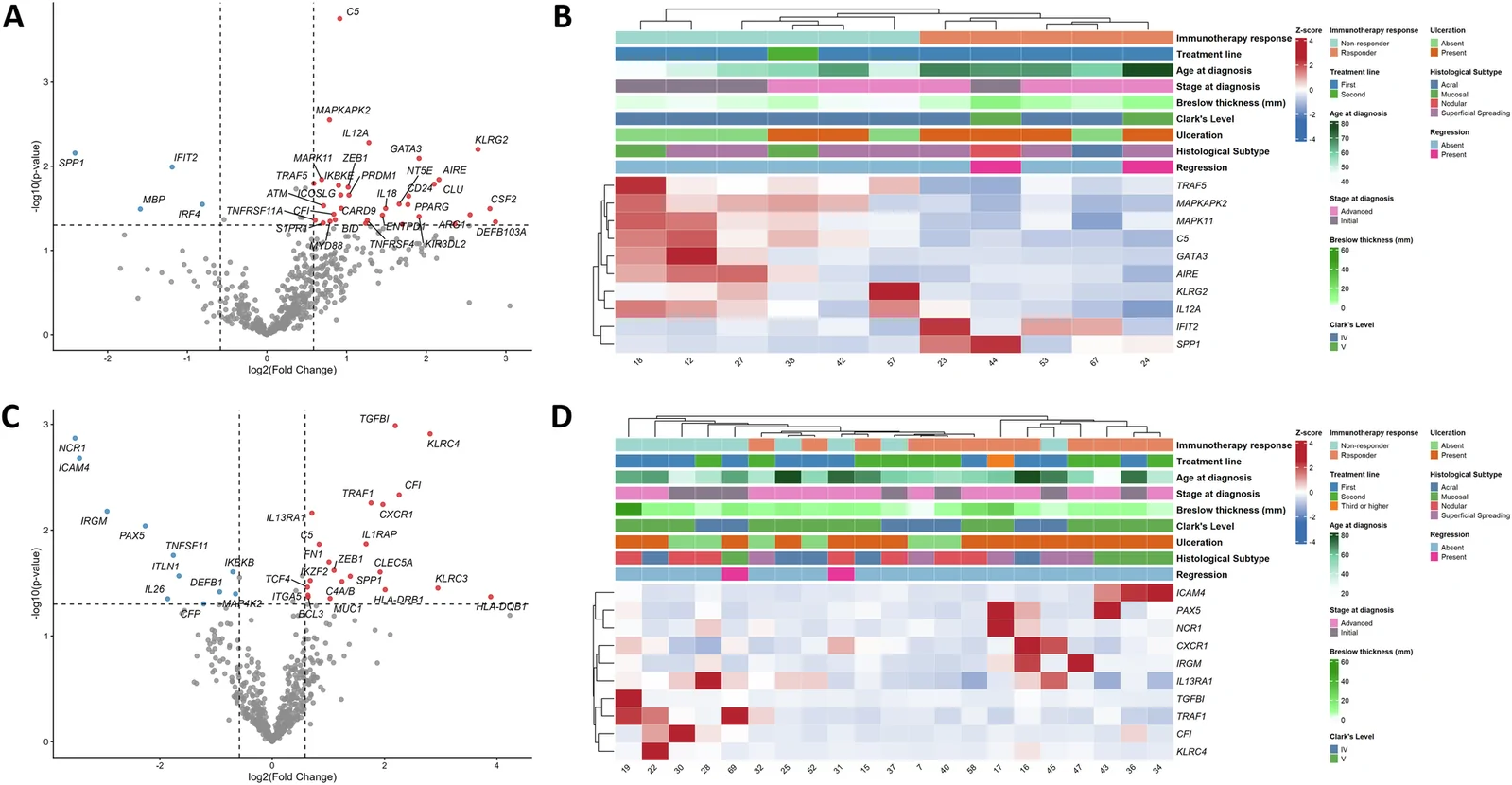
Differentially expressed genes associated with immunotherapy response in brisk and non-brisk melanomas. **A** Volcano plot of genes differentially expressed between immunotherapy responders and non-responders in the brisk subgroup. Red dots indicate genes upregulated in non-responders and blue dots indicate genes upregulated in responders. **B** Heatmap of the most differentially expressed genes in the brisk subgroup annotated by clinicopathological variables. **C** Volcano plot of genes differentially expressed between responders and non-responders in the non-brisk subgroup. Red dots indicate genes upregulated in non-responders and blue dots indicate genes upregulated in responders. **D** Heatmap of the most differentially expressed genes in the non-brisk subgroup annotated by clinicopathological variables.

Genes overexpressed according to immunotherapy response within the brisk and non-brisk TIL groups are presented in Fig. 4. Overall, most upregulated genes were observed in non-responders, and the overexpressed genes differed between the brisk and non-brisk groups. *C5*, *CFI*, and *ZEB1* were overexpressed in non-responders in both groups. In contrast, *SPP1* was overexpressed in responder patients within the brisk group, but in non-responder patients within the non-brisk group. Complete differential expression results are provided in Supplementary Table 1G, H. Additionally, in the brisk group, *SPP1* was negatively correlated with lymphocyte trafficking, phagocytosis and degradation, chemokine signaling, TNF family signaling, and cytotoxic cells. In contrast, in the non-brisk group, *SPP1* was positively correlated with lymphocyte trafficking, phagocytosis and degradation, as well as with the absolute and relative proportions of macrophages, while it was negatively correlated with the relative proportions of cytotoxic cells, T_reg_ cells, and T cells (Supplementary Fig. 3).

**Fig. 5:**
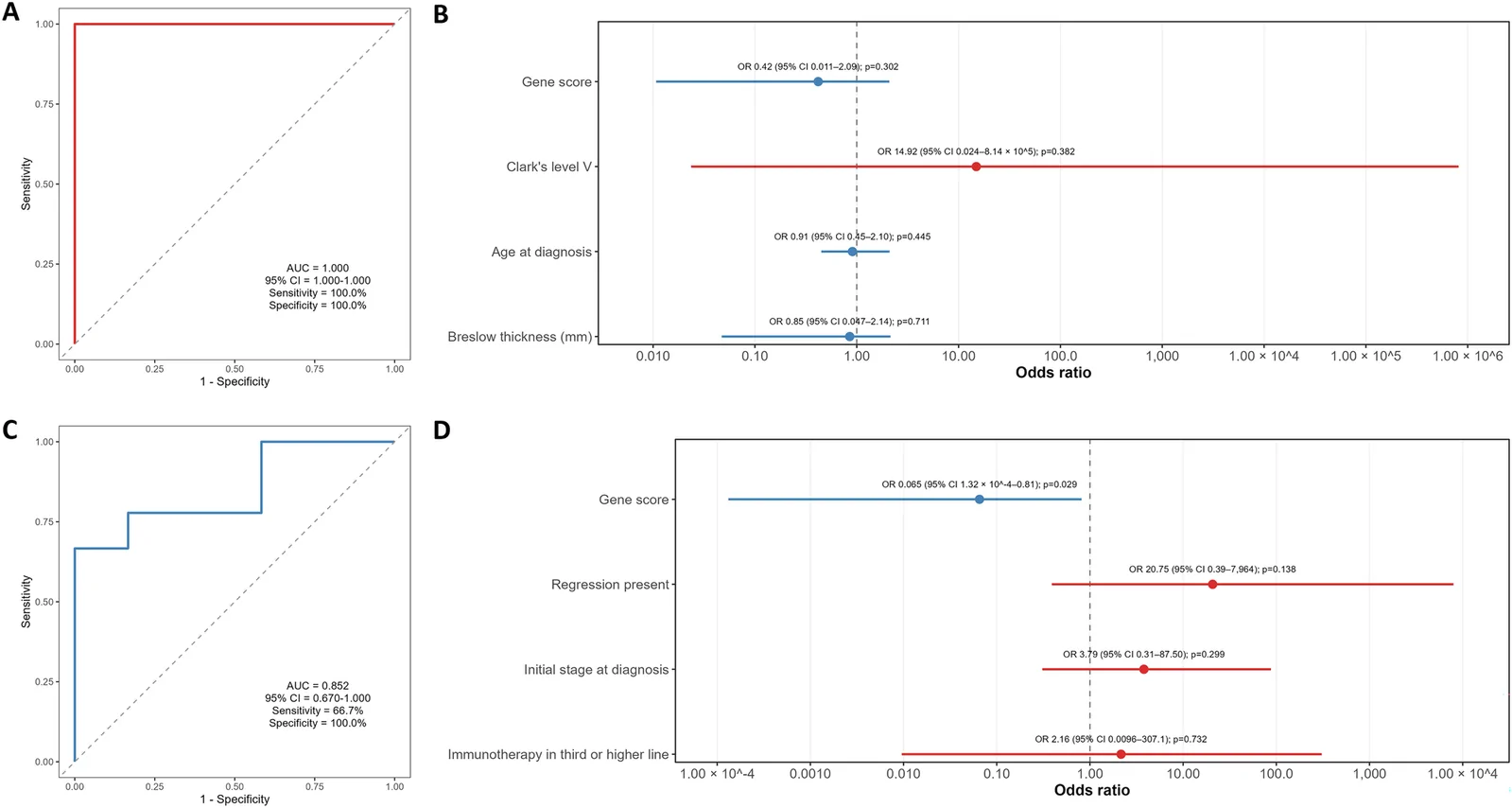
Gene scores for immunotherapy response prediction. **A** Receiver operating characteristic (ROC) curve showing the performance of the 4-gene score for predicting immunotherapy response in the brisk group. **B** Forest plot of the multivariable logistic regression model in the brisk group, including the 4-gene score and clinicopathological covariates. Dots represent odds ratios, and horizontal lines indicate 95% confidence intervals. **C** ROC curve showing the performance of the 3-gene score for predicting immunotherapy response in the non-brisk group. **D** Forest plot of the multivariable logistic regression model in the non-brisk group, including the 3-gene score and clinicopathological covariates. Dots represent odds ratios, and horizontal lines indicate 95% confidence intervals.

The Elastic net penalized logistic regression with response-associated differentially expressed genes resulted in a 4-gene score for the brisk group (*C5*, *MAPKAPK2*, *IL12A*, and *SPP1*) and a 3-gene score for the non-brisk group (*IL13RA1*, *TRAF1*, and *CFI*). Higher scores were found in responders from both groups. The scores showed good performance for predicting immunotherapy response in both the brisk (area under the curve [AUC] = 1.000, Fig. 5A) and non-brisk (AUC = 0.852, Fig. 5C) groups.

Univariate analysis with clinicopathological variables for immunotherapy response is presented in Supplementary Table 1I. After adjustment for clinicopathological predictors of immunotherapy response in multivariate model, the score remained a significant independent predictor only in the non-brisk group (*p* = 0.029), in a model including regression, stage at diagnosis, and line of immunotherapy (Fig. 5D). In the brisk group, the model included Clark level, age at diagnosis, and Breslow thickness (Fig. 5B).

### Upregulated pathways associated with immunotherapy response were different in TILs brisk and non-brisk

Multiple pathways presented higher DGSS in non-responder patients (Supplementary Table 1J, K). In TILs brisk, pathways with higher DGSS in non-responder patients included complement system (DGSS = 1.463), Th1 differentiation (DGSS = 1.442) and Th2 differentiation (DGSS = 1.407). In TILs non-brisk group, genes were more heterogeneous with lower DGSS, with the higher value found in cell adhesion pathway in non-responder patients (DGSS = 0.859).

In the sample-level pathway analysis, no differences were found comparing responder and non-responder patients in TILs brisk and TILS non-brisk cohorts (Supplementary Table 1L, M). Additionally, no differences were found in cell type abundances and cell type ratios (Supplementary Table 1N, O).

### Distinct genes were associated with survival endpoints in TILs brisk and TILS non-brisk cohorts

Genes associated with immunotherapy response were further evaluated for their relationship with survival outcomes. In the brisk cohort, 8 genes were associated with OS, 1 gene with PFS, and 7 genes with PIS (Supplementary Table 1P). In the non-brisk cohort, 4 genes were associated with OS, 6 genes with PFS, and 5 genes with PIS (Supplementary Table 1Q). Considering the genes included in the predictive scores, only *MAPKAPK2* showed a significant association with OS (HR 2.92, 95% CI 1.08–7.90, *p* = 0.035) and PIS (HR 2.41, 95% CI 1.00–5.78, *p* = 0.049).

**Fig. 6:**
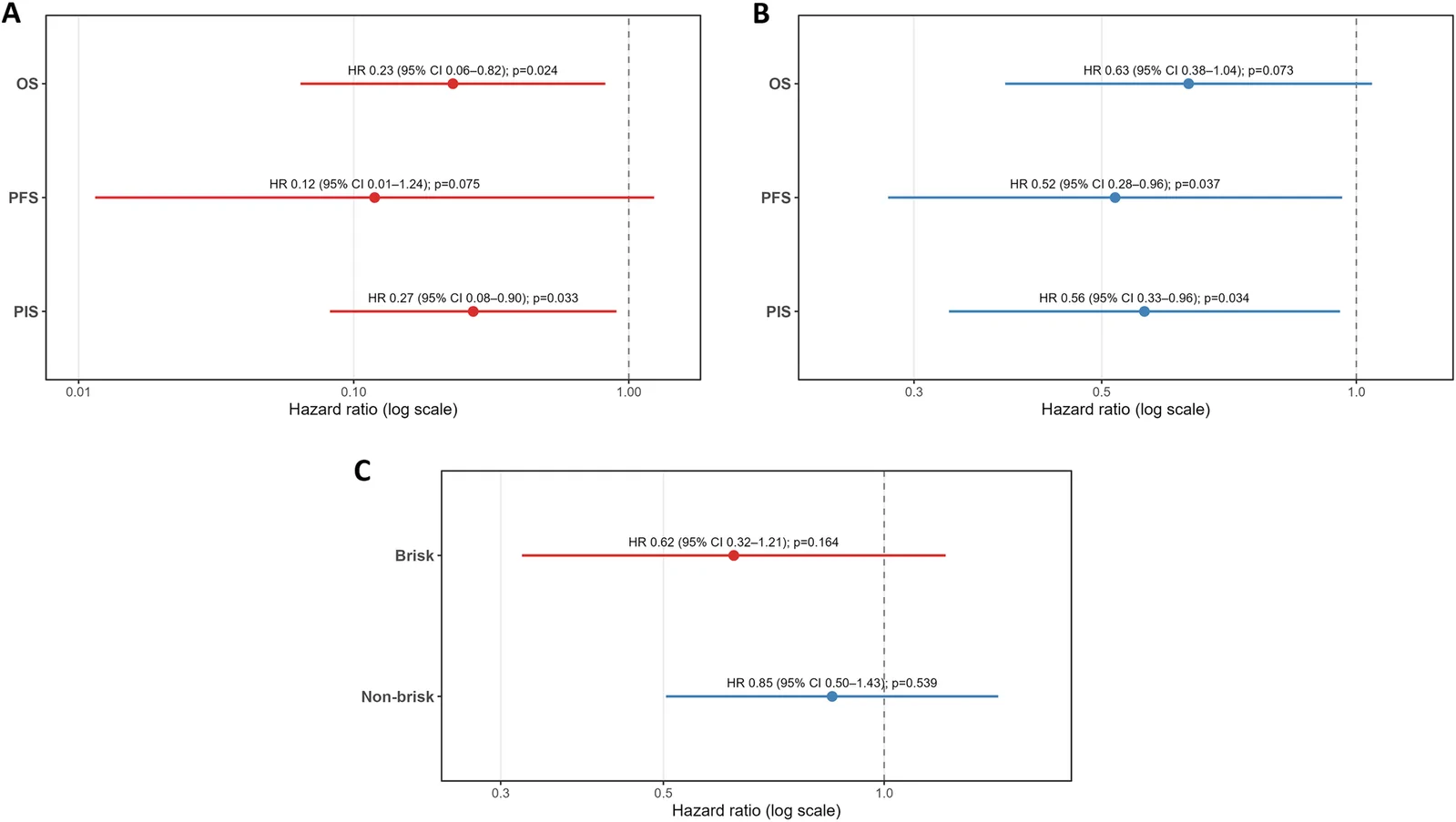
Prognostic value of gene score. **A** Forest plot showing the association of the 4-gene score with overall survival (OS), progression-free survival (PFS), and post-immunotherapy survival (PIS) in the brisk group. **B** Forest plot showing the association of the 3-gene score with OS, PFS, and PIS in the non-brisk group. **C** External validation in the TCGA-SKCM cohort showing the association of the brisk and non-brisk gene scores with OS. Dots represent hazard ratios, and horizontal lines indicate 95% confidence intervals.

In the brisk group, the 4-gene score was also significantly associated with OS (*p* = 0.024) and PIS (*p* = 0.033) (Fig. 6A). In the non-brisk group, the 3-gene score was significantly associated with PFS (*p* = 0.037) and PIS (*p* = 0.034) (Fig. 6B). In both cohorts, higher scores were associated with higher OS, PFS, and PIS. However, in the TCGA cohort, although both scores showed the same trend toward improved OS in the brisk and non-brisk groups, these associations did not reach statistical significance (Fig. 6C).

## Discussion

Histologically, TILs brisk are considered a favorable prognostic indicator. Patients whose melanomas exhibit brisk lymphocytic infiltration often demonstrate better outcomes, including improved overall survival and reduced risk of metastasis, compared to those with non-brisk or absent TILs [17].

In this study, brisk tumors were associated with lower Breslow thickness and lower Clark level (both *p* < 0.01). This finding is consistent with previous studies showing that higher TIL grade is associated with thinner melanomas and with lower Clark level, whereas Breslow thickness and Clark level are established indicators of tumor progression and prognosis in primary cutaneous melanoma [18, 19].

Our analysis identified several differentially expressed genes across TILs pattern. Cross-referencing with the initial discovery cohort supported 22 of these genes as also differentially expressed in the TCGA cohort. From those, *ZAP70* remained significantly expressed in brisk tumors after multiple-testing correcting. *ZAP70* is central to T-cell receptor signaling, which could be directly associated with brisk TILs pattern [13, 20]. On the other hand, *CXCL1* was upregulated in brisk TILs in the internal cohort, while overexpressed in non-brisk TILs in TCGA. The opposite *CXCL1* pattern may reflect different in cellular admixture, considering that *CXCL1* have been associated with non-T-cell inflamed microenvironment [21, 22], which could reflect a distinct microenvironment composition in internal and external cohorts. Sample size limitation should also be considered in the interpretation.

The pathway and cell-composition analyses refine this interpretation. Brisk tumors were enriched for antigen-presentation, T-cell receptor, chemokine, interferon-γ, and lymphocyte-activation programs, together with higher scores for several lymphoid and cytotoxic populations. In contrast, non-brisk tumors showed relatively higher exhausted CD8/TIL, macrophage/TIL, and NK/TIL ratios, consistent with a microenvironment that is not simply less inflamed, but functionally less effective. Recent melanoma studies indicate that immune exclusion can be sustained by tumor-intrinsic resistance programs and by peri-tumoral immune compartments that fail to translate immune presence into productive tumor-cell engagement [21–24]. Our data fits well with this framework and suggests that the histologic brisk/non-brisk distinction reflects different immune topologies rather than only different immune cell quantities.

Applying gene expression analysis to clinical outcomes, we found that the patterns of differentially expressed genes related to immunotherapy response differ between the TILs brisk and TILs non-brisk groups. This indicates that the biological determinants of response are not shared across TIL architectures and supports stratified biomarker development according to baseline immune context.

As a key example, *SPP1* was overexpressed in responder patients with a TILs brisk pattern and in non-responder patients with a TILs non-brisk pattern. SPP1/osteopontin is a pleiotropic matricellular mediator whose biological significance appears to be highly context dependent, varying according to its cellular source and the immune architecture of the tumor microenvironment. Across multiple tumor types, SPP1 is commonly enriched in macrophage-dominated, matrix-remodeling niches and signals through CD44 and integrins to promote immune tolerance, stromal reprogramming, and therapeutic resistance. Mechanistically, SPP1 can function as an immune checkpoint-like signal that suppresses CD8+ T-cell activation, and recent experimental data further show that macrophage-derived SPP1 restrains CD8^+^ T-cell recruitment by limiting CXCL9/CXCL10-associated trafficking, thereby reducing sensitivity to PD-1 blockade [25–28].

In the non-brisk group, high SPP1 in non-responders, together with higher macrophage/phagocytosis-related signals and lower cytotoxic lymphocyte representation, is consistent with an immune-excluded, myeloid-skewed microenvironment, in line with recent studies showing that SPP1^+^ macrophages can cooperate with stromal programs to impair effective CD8^+^ T-cell infiltration and promote resistance to immunotherapy [25, 28, 29].

By contrast, in brisk tumors, the association of high SPP1 with responders suggests that SPP1 should not be interpreted as uniformly immunosuppressive. In the brisk group, which is enriched in TILs, the negative correlation of SPP1 with lymphocyte trafficking, phagocytosis/degradation, TNF and chemokine signaling, as well as with cytotoxic cells, suggests that tumors with high effector T-cell infiltration follow an inflammatory and cytotoxic program that is less dependent on SPP1/TAM axes. Within this already inflamed context, higher SPP1 expression in responders may reflect the presence of specific SPP1⁺ macrophage subsets with a strong capacity to interact with PD-1⁺ CD8 T cells and to support response to checkpoint blockade, as described for SPP1⁺SIRPα⁺ TAMs in patients sensitive to PD-1/PD-L1 blockade [30–32].

To improve the prediction of immunotherapy response, gene-expression scores were developed separately for the brisk and non-brisk cohorts. Overall, these scores appear to capture the complex interplay between effective antitumor immunity and counter-regulatory inflammatory programs and differ according to pattern of lymphocyte infiltration. This interpretation is supported by the fact that some score components, such as *IL13RA1* in non-brisk tumors, are more consistent with an immunosuppressive microenvironment, whereas others, such as *IL12A* in brisk tumors, are classically linked to pro-inflammatory immune activation and may instead reflect compensatory or adaptive responses [33–37]. Notably, these composite scores improved the stratification of patients according to treatment response, particularly in non-brisk tumors, where the score remained an independent predictor after multivariable adjustment.

Finally, the survival analyses further support the clinical relevance of these signatures beyond the prediction of binary response. The concordance between treatment response and survival outcomes reinforces the interpretation that these scores capture biologically meaningful, treatment-sensitive tumor states. Although the TCGA cohort showed the same directional trend, the lack of statistical significance should be interpreted with caution rather than as definitive external validation. Importantly, TCGA-SKCM was established largely before the routine use of immune checkpoint inhibitors; therefore, these scores still require validation in independent cohorts of patients uniformly treated with immunotherapy.

Overall, our data supports the concept that TIL brisk morphology aligns with broader immune-associated transcriptional programs, while outcome relevant heterogeneity persists within both brisk and non-brisk tumors. Importantly, these findings should be interpreted as exploratory and hypothesis-generating given key limitations: this is a bulk gene expression analysis in a small cohort, with reduced statistical power and increased uncertainty in secondary analyses after stratification into brisk and non-brisk subgroups. Bulk profiling also cannot resolve cell-type–specific versus tumor-intrinsic contributions or spatial organization of immune cells, which are likely to influence pathway activity and clinical associations. Accordingly, negative or non-significant findings—particularly in subgroup and external validation analyses—should not be interpreted as evidence of absence, as the modest sample size may have limited the power to detect effects of small-to-moderate magnitude. Rather, these results should be considered inconclusive and hypothesis-generating, warranting confirmation in larger and more homogeneous cohorts.

In addition, because molecular analyses were performed on primary melanoma samples whereas treatment response was assessed in the advanced setting, our results must be interpreted considering the known temporal and site-specific heterogeneity of the melanoma immune microenvironment. Although major mutational drivers are often conserved during progression [38], immune biomarkers such as PD-L1 expression and lymphocyte infiltration can vary between primary and metastatic lesions and across metastatic sites [39–41]. Thus, primary-tumor TIL-related transcriptional features may capture a biologically meaningful baseline immune context but may incompletely reflect the metastatic immune microenvironment present at treatment initiation.

The interpretation of our results should also consider the inclusion of different histological subtypes. Mucosal and acral melanomas have been reported to differ from conventional cutaneous melanoma in their immune microenvironment and in their responsiveness to immune checkpoint inhibitors [42–46]. In the present study, no significant differences were observed according to histological subtype. However, this should not be interpreted as evidence of biological equivalence, since the limited sample size may have reduced the power to detect subtype-related differences. Therefore, the potential impact of histological subtypes should be further evaluated in larger cohorts.

Accordingly, validation in larger, independent cohorts and with higher-resolution approaches (e.g., spatial or single-cell profiling) could contribute to additional information regarding prognostic significance of TILs in melanoma patients treated with anti-PD-1.

## Supplementary information

Supplementary Figures Captions

Supplementary Tables

Supplementary Figure 3

Supplementary Figure 1

Supplementary Figure 2

## Data Availability

The datasets generated during and/or analyzed during the current study are available from the corresponding author on reasonable request.
