# Supplementary material for: The tumoural landscape of lymphocytes and immune pathways in immunotherapy-treated melanoma patients
Source: Genes Immun. 2026 Jul 15;27(5):506–15. doi: 10.1038/s41435-026-00406-1 (PMC13658024; doi:10.1038/s41435-026-00406-1)
Supplement: Supplementary file 1 [file 41435_2026_406_MOESM1_ESM.docx]

**SUPPLEMENTARY FIGURES LEGENDS**

**Supplementary Figure 1.** Complete analysis of pathway score distributions comparing brisk and non-brisk groups (Pathway scoring module).

**Supplementary Figure 2.** Immune cell type scores without statistical significance, including individual (A) cell types and (B) cell ratios.

**Supplementary Figure 3.** Correlation of *SPP1* gene expression and pathways, cell type (relative) and cell type (raw) scores in brisk and non-brisk groups.
