# Supplementary material for: The tumoural landscape of lymphocytes and immune pathways in immunotherapy-treated melanoma patients
Source: Genes Immun. 2026 Jul 15;27(5):506–15. doi: 10.1038/s41435-026-00406-1 (PMC13658024; doi:10.1038/s41435-026-00406-1)
Supplement: Supplementary file 2 [file 41435_2026_406_MOESM2_ESM.docx]

| **Supplementary Table 1A.** Gene list of Human Immunology v2 panel. | |
| --- | --- |
| **Gene symbol** | **Gene name** |
| *ABCB1* | ATP-binding cassette, sub-family B (MDR/TAP), member 1 |
| *ABL1* | c-abl oncogene 1, non-receptor tyrosine kinase |
| *ADA* | adenosine deaminase |
| *AHR* | aryl hydrocarbon receptor |
| *AICDA* | activation-induced cytidine deaminase |
| *AIRE* | autoimmune regulator |
| *APP* | amyloid beta (A4) precursor protein |
| *ARG1* | arginase, liver |
| *ARG2* | arginase, type II |
| *ARHGDIB* | Rho GDP dissociation inhibitor (GDI) beta |
| *ATG10* | ATG10 autophagy related 10 homolog (S. cerevisiae) |
| *ATG12* | ATG12 autophagy related 12 homolog (S. cerevisiae) |
| *ATG16L1* | ATG16 autophagy related 16-like 1 (S. cerevisiae) |
| *ATG5* | ATG5 autophagy related 5 homolog (S. cerevisiae) |
| *ATG7* | ATG7 autophagy related 7 homolog (S. cerevisiae) |
| *ATM* | ataxia telangiectasia mutated |
| *B2M* | beta-2-microglobulin |
| *B3GAT1* | beta-1,3-glucuronyltransferase 1 (glucuronosyltransferase P) |
| *BATF* | basic leucine zipper transcription factor, ATF-like |
| *BATF3* | basic leucine zipper transcription factor, ATF-like 3 |
| *BAX* | BCL2-associated X protein |
| *BCAP31* | B-cell receptor-associated protein 31 |
| *BCL10* | B-cell CLL/lymphoma 10 |
| *BCL2* | B-cell CLL/lymphoma 2 |
| *BCL2L11* | BCL2-like 11 (apoptosis facilitator) |
| *BCL3* | B-cell CLL/lymphoma 3 |
| *BCL6* | B-cell CLL/lymphoma 6 |
| *BID* | BH3 interacting domain death agonist |
| *BLNK* | B-cell linker |
| *BST1* | bone marrow stromal cell antigen 1 |
| *BST2* | bone marrow stromal cell antigen 2 |
| *BTK* | Bruton agammaglobulinemia tyrosine kinase |
| *BTLA* | B and T lymphocyte associated |
| *C14orf166* | chromosome 14 open reading frame 166 |
| *C1QA* | complement component 1, q subcomponent, A chain |
| *C1QB* | complement component 1, q subcomponent, B chain |
| *C1QBP* | complement component 1, q subcomponent binding protein |
| *C1R* | complement component 1, r subcomponent |
| *C1S* | complement component 1, s subcomponent |
| *C2* | complement component 2 |
| *C3* | complement component 3 |
| *C4A/B* | complement component 4A (Rodgers blood group)/complement component 4B (Chido blood group) |
| *C4BPA* | complement component 4 binding protein, alpha |
| *C5* | complement component 5 |
| *C6* | complement component 6 |
| *C7* | complement component 7 |
| *C8A* | complement component 8, alpha polypeptide |
| *C8B* | complement component 8, beta polypeptide |
| *C8G* | complement component 8, gamma polypeptide |
| *C9* | complement component 9 |
| *CAMP* | cathelicidin antimicrobial peptide |
| *CARD9* | caspase recruitment domain family, member 9 |
| *CASP1* | caspase 1, apoptosis-related cysteine peptidase (interleukin 1, beta, convertase) |
| *CASP10* | caspase 10, apoptosis-related cysteine peptidase |
| *CASP2* | caspase 2, apoptosis-related cysteine peptidase |
| *CASP3* | caspase 3, apoptosis-related cysteine peptidase |
| *CASP8* | caspase 8, apoptosis-related cysteine peptidase |
| *CCBP2* | chemokine binding protein 2 |
| *CCL11* | chemokine (C-C motif) ligand 11 |
| *CCL13* | chemokine (C-C motif) ligand 13 |
| *CCL15* | chemokine (C-C motif) ligand 15 |
| *CCL16* | chemokine (C-C motif) ligand 16 |
| *CCL18* | chemokine (C-C motif) ligand 18 (pulmonary and activation-regulated) |
| *CCL19* | chemokine (C-C motif) ligand 19 |
| *CCL2* | chemokine (C-C motif) ligand 2 |
| *CCL20* | chemokine (C-C motif) ligand 20 |
| *CCL22* | chemokine (C-C motif) ligand 22 |
| *CCL23* | chemokine (C-C motif) ligand 23 |
| *CCL24* | chemokine (C-C motif) ligand 24 |
| *CCL26* | chemokine (C-C motif) ligand 26 |
| *CCL3* | chemokine (C-C motif) ligand 3 |
| *CCL4* | chemokine (C-C motif) ligand 4 |
| *CCL5* | chemokine (C-C motif) ligand 5 |
| *CCL7* | chemokine (C-C motif) ligand 7 |
| *CCL8* | chemokine (C-C motif) ligand 8 |
| *CCND3* | cyclin D3 |
| *CCR1* | chemokine (C-C motif) receptor 1 |
| *CCR10* | chemokine (C-C motif) receptor 10 |
| *CCR2* | chemokine (C-C motif) receptor 2 |
| *CCR5* | chemokine (C-C motif) receptor 5 |
| *CCR6* | chemokine (C-C motif) receptor 6 |
| *CCR7* | chemokine (C-C motif) receptor 7 |
| *CCR8* | chemokine (C-C motif) receptor 8 |
| *CCRL1* | chemokine (C-C motif) receptor-like 1 |
| *CCRL2* | chemokine (C-C motif) receptor-like 2 |
| *CD14* | CD14 molecule |
| *CD160* | CD160 molecule |
| *CD163* | CD163 molecule |
| *CD164* | CD164 molecule, sialomucin |
| *CD19* | CD19 molecule |
| *CD1A* | CD1a molecule |
| *CD1D* | CD1d molecule |
| *CD2* | CD2 molecule |
| *CD209* | CD209 molecule |
| *CD22* | CD22 molecule |
| *CD24* | CD24 molecule |
| *CD244* | CD244 molecule, natural killer cell receptor 2B4 |
| *CD247* | CD247 molecule |
| *CD27* | CD27 molecule |
| *CD274* | CD274 molecule |
| *CD276* | CD276 molecule |
| *CD28* | CD28 molecule |
| *CD34* | CD34 molecule |
| *CD36* | CD36 molecule (thrombospondin receptor) |
| *CD3D* | CD3d molecule, delta (CD3-TCR complex) |
| *CD3E* | CD3e molecule, epsilon (CD3-TCR complex) |
| *CD3EAP* | CD3e molecule, epsilon associated protein |
| *CD4* | CD4 molecule |
| *CD40* | CD40 molecule, TNF receptor superfamily member 5 |
| *CD40LG* | CD40 ligand |
| *CD44* | CD44 molecule (Indian blood group) |
| *CD46* | CD46 molecule, complement regulatory protein |
| *CD48* | CD48 molecule |
| *CD5* | CD5 molecule |
| *CD53* | CD53 molecule |
| *CD55* | CD55 molecule, decay accelerating factor for complement (Cromer blood group) |
| *CD58* | CD58 molecule |
| *CD59* | CD59 molecule, complement regulatory protein |
| *CD6* | CD6 molecule |
| *CD7* | CD7 molecule |
| *CD70* | CD70 molecule |
| *CD74* | CD74 molecule, major histocompatibility complex, class II invariant chain |
| *CD79A* | CD79a molecule, immunoglobulin-associated alpha |
| *CD79B* | CD79b molecule, immunoglobulin-associated beta |
| *CD80* | CD80 molecule |
| *CD81* | CD81 molecule |
| *CD82* | CD82 molecule |
| *CD83* | CD83 molecule |
| *CD86* | CD86 molecule |
| *CD8A* | CD8a molecule |
| *CD8B* | CD8b molecule |
| *CD9* | CD9 molecule |
| *CD96* | CD96 molecule |
| *CD97* | CD97 molecule |
| *CD99* | CD99 molecule |
| *CDH5* | cadherin 5, type 2 (vascular endothelium) |
| *CDKN1A* | cyclin-dependent kinase inhibitor 1A (p21, Cip1) |
| *CEACAM1* | carcinoembryonic antigen-related cell adhesion molecule 1 (biliary glycoprotein) |
| *CEACAM6* | carcinoembryonic antigen-related cell adhesion molecule 6 (non-specific cross reacting antigen) |
| *CEACAM8* | carcinoembryonic antigen-related cell adhesion molecule 8 |
| *CEBPB* | CCAAT/enhancer binding protein (C/EBP), beta |
| *CFB* | complement factor B |
| *CFD* | complement factor D (adipsin) |
| *CFH* | complement factor H |
| *CFI* | complement factor I |
| *CFP* | complement factor properdin |
| *CHUK* | conserved helix-loop-helix ubiquitous kinase |
| *CIITA* | class II, major histocompatibility complex, transactivator |
| *CISH* | cytokine inducible SH2-containing protein |
| *CLEC4A* | C-type lectin domain family 4, member A |
| *CLEC4E* | C-type lectin domain family 4, member E |
| *CLEC5A* | C-type lectin domain family 5, member A |
| *CLEC6A* | C-type lectin domain family 6, member A |
| *CLEC7A* | C-type lectin domain family 7, member A |
| *CLU* | clusterin |
| *CMKLR1* | chemokine-like receptor 1 |
| *CR1* | complement component (3b/4b) receptor 1 (Knops blood group) |
| *CR2* | complement component (3d/Epstein Barr virus) receptor 2 |
| *CRADD* | CASP2 and RIPK1 domain containing adaptor with death domain |
| *CSF1* | colony stimulating factor 1 (macrophage) |
| *CSF1R* | colony stimulating factor 1 receptor |
| *CSF2* | colony stimulating factor 2 (granulocyte-macrophage) |
| *CSF2RB* | colony stimulating factor 2 receptor, beta, low-affinity (granulocyte-macrophage) |
| *CSF3R* | colony stimulating factor 3 receptor (granulocyte) |
| *CTLA4_all* | cytotoxic T-lymphocyte-associated protein 4 |
| *CTLA4-TM* | cytotoxic T-lymphocyte-associated protein 4 |
| *sCTLA4* | cytotoxic T-lymphocyte-associated protein 4 |
| *CTNNB1* | catenin (cadherin-associated protein), beta 1, 88kDa |
| *CTSC* | cathepsin C |
| *CTSG* | cathepsin G |
| *CTSS* | cathepsin S |
| *CUL9* | cullin 9 |
| *CX3CL1* | chemokine (C-X3-C motif) ligand 1 |
| *CX3CR1* | chemokine (C-X3-C motif) receptor 1 |
| *CXCL1* | chemokine (C-X-C motif) ligand 1 (melanoma growth stimulating activity, alpha) |
| *CXCL10* | chemokine (C-X-C motif) ligand 10 |
| *CXCL11* | chemokine (C-X-C motif) ligand 11 |
| *CXCL12* | chemokine (C-X-C motif) ligand 12 |
| *CXCL13* | chemokine (C-X-C motif) ligand 13 |
| *CXCL2* | chemokine (C-X-C motif) ligand 2 |
| *CXCL9* | chemokine (C-X-C motif) ligand 9 |
| *CXCR1* | chemokine (C-X-C motif) receptor 1 |
| *CXCR2* | chemokine (C-X-C motif) receptor 2 |
| *CXCR3* | chemokine (C-X-C motif) receptor 3 |
| *CXCR4* | chemokine (C-X-C motif) receptor 4 |
| *CXCR6* | chemokine (C-X-C motif) receptor 6 |
| *CYBB* | cytochrome b-245, beta polypeptide |
| *DEFB1* | defensin, beta 1 |
| *DEFB103A* | defensin, beta 103A |
| *DEFB103B* | defensin, beta 103B |
| *DEFB4A* | defensin, beta 4A |
| *DPP4* | dipeptidyl-peptidase 4 |
| *DUSP4* | dual specificity phosphatase 4 |
| *EBI3* | Epstein-Barr virus induced 3 |
| *EDNRB* | endothelin receptor type B |
| *EGR1* | early growth response 1 |
| *EGR2* | early growth response 2 |
| *ENTPD1* | ectonucleoside triphosphate diphosphohydrolase 1 |
| *EOMES* | eomesodermin |
| *ETS1* | v-ets erythroblastosis virus E26 oncogene homolog 1 (avian) |
| *FADD* | Fas (TNFRSF6)-associated via death domain |
| *FAS* | Fas (TNF receptor superfamily, member 6) |
| *FCAR* | Fc fragment of IgA, receptor for |
| *FCER1A* | Fc fragment of IgE, high affinity I, receptor for; alpha polypeptide |
| *FCER1G* | Fc fragment of IgE, high affinity I, receptor for; gamma polypeptide |
| *FCGR1A/B* | Fc fragment of IgG, high affinity Ia, receptor (CD64)/Fc fragment of IgG, high affinity Ib, receptor (CD64) |
| *FCGR2A* | Fc fragment of IgG, low affinity IIa, receptor (CD32) |
| *FCGR2A/C* | Fc fragment of IgG, low affinity IIa, receptor (CD32)/Fc fragment of IgG, low affinity IIc, receptor for (CD32) |
| *FCGR2B* | Fc fragment of IgG, low affinity IIb, receptor (CD32) |
| *FCGR3A/B* | Fc fragment of IgG, low affinity IIIa, receptor (CD16a)/Fc fragment of IgG, low affinity IIIb, receptor (CD16a) |
| *FCGRT* | Fc fragment of IgG, receptor, transporter, alpha |
| *FKBP5* | FK506 binding protein 5 |
| *FN1* | fibronectin 1 |
| *FOXP3* | forkhead box P3 |
| *FYN* | FYN oncogene related to SRC, FGR, YES |
| *GATA3* | GATA binding protein 3 |
| *GBP1* | guanylate binding protein 1, interferon-inducible |
| *GBP5* | guanylate binding protein 5 |
| *GFI1* | growth factor independent 1 transcription repressor |
| *GNLY* | granulysin |
| *GP1BB* | glycoprotein Ib (platelet), beta polypeptide |
| *GPI* | glucose-6-phosphate isomerase |
| *GPR183* | G protein-coupled receptor 183 |
| *GZMA* | granzyme A (granzyme 1, cytotoxic T-lymphocyte-associated serine esterase 3) |
| *GZMB* | granzyme B (granzyme 2, cytotoxic T-lymphocyte-associated serine esterase 1) |
| *GZMK* | granzyme K (granzyme 3; tryptase II) |
| *HAMP* | hepcidin antimicrobial peptide |
| *HAVCR2* | hepatitis A virus cellular receptor 2 |
| *HFE* | hemochromatosis |
| *HLA-A* | major histocompatibility complex, class I, A |
| *HLA-B* | major histocompatibility complex, class I, B |
| *HLA-C* | major histocompatibility complex, class I, C |
| *HLA-DMA* | major histocompatibility complex, class II, DM alpha |
| *HLA-DMB* | major histocompatibility complex, class II, DM beta |
| *HLA-DOB* | major histocompatibility complex, class II, DO beta |
| *HLA-DPA1* | major histocompatibility complex, class II, DP alpha 1 |
| *HLA-DPB1* | major histocompatibility complex, class II, DP beta 1 |
| *HLA-DQA1* | major histocompatibility complex, class II, DQ alpha 1 |
| *HLA-DQB1* | major histocompatibility complex, class II, DQ beta 1 |
| *HLA-DRA* | major histocompatibility complex, class II, DR alpha |
| *HLA-DRB1* | major histocompatibility complex, class II, DR beta 1 |
| *HLA-DRB3* | major histocompatibility complex, class II, DR beta 3 |
| *HRAS* | v-Ha-ras Harvey rat sarcoma viral oncogene homolog |
| *ICAM1* | intercellular adhesion molecule 1 |
| *ICAM2* | intercellular adhesion molecule 2 |
| *ICAM3* | intercellular adhesion molecule 3 |
| *ICAM4* | intercellular adhesion molecule 4 (Landsteiner-Wiener blood group) |
| *ICAM5* | intercellular adhesion molecule 5, telencephalin |
| *ICOS* | inducible T-cell co-stimulator |
| *ICOSLG* | inducible T-cell co-stimulator ligand |
| *IDO1* | indoleamine 2,3-dioxygenase 1 |
| *IFI16* | interferon, gamma-inducible protein 16 |
| *IFI35* | interferon-induced protein 35 |
| *IFIH1* | interferon induced with helicase C domain 1 |
| *IFIT2* | interferon-induced protein with tetratricopeptide repeats 2 |
| *IFITM1* | interferon induced transmembrane protein 1 (9-27) |
| *IFNA1/13* | interferon, alpha 1/interferon, alpha 13 |
| *IFNA2* | interferon, alpha 2 |
| *IFNAR1* | interferon (alpha, beta and omega) receptor 1 |
| *IFNAR2* | interferon (alpha, beta and omega) receptor 2 |
| *IFNB1* | interferon, beta 1, fibroblast |
| *IFNG* | interferon, gamma |
| *IFNGR1* | interferon gamma receptor 1 |
| *IGF2R* | insulin-like growth factor 2 receptor |
| *IKBKAP* | inhibitor of kappa light polypeptide gene enhancer in B-cells, kinase complex-associated protein |
| *IKBKB* | inhibitor of kappa light polypeptide gene enhancer in B-cells, kinase beta |
| *IKBKE* | inhibitor of kappa light polypeptide gene enhancer in B-cells, kinase epsilon |
| *IKBKG* | inhibitor of kappa light polypeptide gene enhancer in B-cells, kinase gamma |
| *IKZF1* | IKAROS family zinc finger 1 (Ikaros) |
| *IKZF2* | IKAROS family zinc finger 2 (Helios) |
| *IKZF3* | IKAROS family zinc finger 3 (Aiolos) |
| *IL10* | interleukin 10 |
| *IL10RA* | interleukin 10 receptor, alpha |
| *IL11RA* | interleukin 11 receptor, alpha |
| *IL12A* | interleukin 12A (natural killer cell stimulatory factor 1, cytotoxic lymphocyte maturation factor 1, p35) |
| *IL12B* | interleukin 12B (natural killer cell stimulatory factor 2, cytotoxic lymphocyte maturation factor 2, p40) |
| *IL12RB1* | interleukin 12 receptor, beta 1 |
| *IL13* | interleukin 13 |
| *IL13RA1* | interleukin 13 receptor, alpha 1 |
| *IL15* | interleukin 15 |
| *IL16* | interleukin 16 |
| *IL17A* | interleukin 17A |
| *IL17B* | interleukin 17B |
| *IL17F* | interleukin 17F |
| *IL18* | interleukin 18 (interferon-gamma-inducing factor) |
| *IL18R1* | interleukin 18 receptor 1 |
| *IL18RAP* | interleukin 18 receptor accessory protein |
| *IL19* | interleukin 19 |
| *IL1A* | interleukin 1, alpha |
| *IL1B* | interleukin 1, beta |
| *IL1R1* | interleukin 1 receptor, type I |
| *IL1R2* | interleukin 1 receptor, type II |
| *IL1RAP* | interleukin 1 receptor accessory protein |
| *IL1RL1* | interleukin 1 receptor-like 1 |
| *IL1RL2* | interleukin 1 receptor-like 2 |
| *IL1RN* | interleukin 1 receptor antagonist |
| *IL2* | interleukin 2 |
| *IL20* | interleukin 20 |
| *IL21* | interleukin 21 |
| *IL21R* | interleukin 21 receptor |
| *IL22* | interleukin 22 |
| *IL22RA2* | interleukin 22 receptor, alpha 2 |
| *IL23A* | interleukin 23, alpha subunit p19 |
| *IL23R* | interleukin 23 receptor |
| *IL26* | interleukin 26 |
| *IL27* | interleukin 27 |
| *IL28A* | interleukin 28A (interferon, lambda 2) |
| *IL28A/B* | interleukin 28A (interferon, lambda 2)/interleukin 28B (interferon, lambda 3) |
| *IL29* | interleukin 29 (interferon, lambda 1) |
| *IL2RA* | interleukin 2 receptor, alpha |
| *IL2RB* | interleukin 2 receptor, beta |
| *IL2RG* | interleukin 2 receptor, gamma |
| *IL3* | interleukin 3 (colony-stimulating factor, multiple) |
| *IL32* | interleukin 32 |
| *IL4* | interleukin 4 |
| *IL4R* | interleukin 4 receptor |
| *IL5* | interleukin 5 (colony-stimulating factor, eosinophil) |
| *IL6* | interleukin 6 (interferon, beta 2) |
| *IL6R* | interleukin 6 receptor |
| *IL6ST* | interleukin 6 signal transducer (gp130, oncostatin M receptor) |
| *IL7* | interleukin 7 |
| *IL7R* | interleukin 7 receptor |
| *IL8* | interleukin 8 |
| *IL9* | interleukin 9 |
| *ILF3* | interleukin enhancer binding factor 3, 90kDa |
| *IRAK1* | interleukin-1 receptor-associated kinase 1 |
| *IRAK2* | interleukin-1 receptor-associated kinase 2 |
| *IRAK3* | interleukin-1 receptor-associated kinase 3 |
| *IRAK4* | interleukin-1 receptor-associated kinase 4 |
| *IRF1* | interferon regulatory factor 1 |
| *IRF3* | interferon regulatory factor 3 |
| *IRF4* | interferon regulatory factor 4 |
| *IRF5* | interferon regulatory factor 5 |
| *IRF7* | interferon regulatory factor 7 |
| *IRF8* | interferon regulatory factor 8 |
| *IRGM* | immunity-related GTPase family, M |
| *ITGA2B* | integrin, alpha 2b (platelet glycoprotein IIb of IIb/IIIa complex, antigen CD41) |
| *ITGA4* | integrin, alpha 4 (antigen CD49D, alpha 4 subunit of VLA-4 receptor) |
| *ITGA5* | integrin, alpha 5 (fibronectin receptor, alpha polypeptide) |
| *ITGA6* | integrin, alpha 6 |
| *ITGAE* | integrin, alpha E (antigen CD103, human mucosal lymphocyte antigen 1; alpha polypeptide) |
| *ITGAL* | integrin, alpha L (antigen CD11A (p180), lymphocyte function-associated antigen 1; alpha polypeptide) |
| *ITGAM* | integrin, alpha M (complement component 3 receptor 3 subunit) |
| *ITGAX* | integrin, alpha X (complement component 3 receptor 4 subunit) |
| *ITGB1* | integrin, beta 1 (fibronectin receptor, beta polypeptide, antigen CD29 includes MDF2, MSK12) |
| *ITGB2* | integrin, beta 2 (complement component 3 receptor 3 and 4 subunit) |
| *ITLN1* | intelectin 1 (galactofuranose binding) |
| *ITLN2* | intelectin 2 |
| *JAK1* | Janus kinase 1 |
| *JAK2* | Janus kinase 2 |
| *JAK3* | Janus kinase 3 |
| *KCNJ2* | potassium inwardly-rectifying channel, subfamily J, member 2 |
| *KIR_Activating_Subgroup_1* | killer cell immunoglobulin-like receptor |
| *KIR_Activating_Subgroup_2* | killer cell immunoglobulin-like receptor |
| *KIR_Inhibiting_Subgroup_1* | killer cell immunoglobulin-like receptor |
| *KIR_Inhibiting_Subgroup_2* | killer cell immunoglobulin-like receptor |
| *KIR3DL1* | killer cell immunoglobulin-like receptor, three domains, long cytoplasmic tail, 1 |
| *KIR3DL2* | killer cell immunoglobulin-like receptor, three domains, long cytoplasmic tail, 2 |
| *KIR3DL3* | killer cell immunoglobulin-like receptor, three domains, long cytoplasmic tail, 3 |
| *KIT* | v-kit Hardy-Zuckerman 4 feline sarcoma viral oncogene homolog |
| *KLRAP1* | killer cell lectin-like receptor subfamily A pseudogene 1 |
| *KLRB1* | killer cell lectin-like receptor subfamily B, member 1 |
| *KLRC1* | killer cell lectin-like receptor subfamily C, member 1 |
| *KLRC2* | killer cell lectin-like receptor subfamily C, member 2 |
| *KLRC3* | killer cell lectin-like receptor subfamily C, member 3 |
| *KLRC4* | killer cell lectin-like receptor subfamily C, member 4 |
| *KLRD1* | killer cell lectin-like receptor subfamily D, member 1 |
| *KLRF1* | killer cell lectin-like receptor subfamily F, member 1 |
| *KLRF2* | killer cell lectin-like receptor subfamily F, member 2 |
| *KLRG1* | killer cell lectin-like receptor subfamily G, member 1 |
| *KLRG2* | killer cell lectin-like receptor subfamily G, member 2 |
| *KLRK1* | killer cell lectin-like receptor subfamily K, member 1 |
| *LAG3* | lymphocyte-activation gene 3 |
| *LAIR1* | leukocyte-associated immunoglobulin-like receptor 1 |
| *LAMP3* | lysosomal-associated membrane protein 3 |
| *LCK* | lymphocyte-specific protein tyrosine kinase |
| *LCP2* | lymphocyte cytosolic protein 2 (SH2 domain containing leukocyte protein of 76kDa) |
| *LEF1* | lymphoid enhancer-binding factor 1 |
| *LGALS3* | lectin, galactoside-binding, soluble, 3 |
| *LIF* | leukemia inhibitory factor (cholinergic differentiation factor) |
| *LILRA1* | leukocyte immunoglobulin-like receptor, subfamily A (with TM domain), member 1 |
| *LILRA2* | leukocyte immunoglobulin-like receptor, subfamily A (with TM domain), member 2 |
| *LILRA3* | leukocyte immunoglobulin-like receptor, subfamily A (without TM domain), member 3 |
| *LILRA4* | leukocyte immunoglobulin-like receptor, subfamily A (with TM domain), member 4 |
| *LILRA5* | leukocyte immunoglobulin-like receptor, subfamily A (with TM domain), member 5 |
| *LILRA6* | leukocyte immunoglobulin-like receptor, subfamily A (with TM domain), member 6 |
| *LILRB1* | leukocyte immunoglobulin-like receptor, subfamily B (with TM and ITIM domains), member 1 |
| *LILRB2* | leukocyte immunoglobulin-like receptor, subfamily B (with TM and ITIM domains), member 2 |
| *LILRB3* | leukocyte immunoglobulin-like receptor, subfamily B (with TM and ITIM domains), member 3 |
| *LILRB4* | leukocyte immunoglobulin-like receptor, subfamily B (with TM and ITIM domains), member 4 |
| *LILRB5* | leukocyte immunoglobulin-like receptor, subfamily B (with TM and ITIM domains), member 5 |
| *LITAF* | lipopolysaccharide-induced TNF factor |
| *LTA* | lymphotoxin alpha (TNF superfamily, member 1) |
| *LTB4R* | leukotriene B4 receptor |
| *LTB4R2* | leukotriene B4 receptor 2 |
| *LTBR* | lymphotoxin beta receptor (TNFR superfamily, member 3) |
| *LTF* | lactotransferrin |
| *LY96* | lymphocyte antigen 96 |
| *MAF* | v-maf musculoaponeurotic fibrosarcoma oncogene homolog (avian) |
| *MALT1* | mucosa associated lymphoid tissue lymphoma translocation gene 1 |
| *MAP4K1* | mitogen-activated protein kinase kinase kinase kinase 1 |
| *MAP4K2* | mitogen-activated protein kinase kinase kinase kinase 2 |
| *MAP4K4* | mitogen-activated protein kinase kinase kinase kinase 4 |
| *MAPK1* | mitogen-activated protein kinase 1 |
| *MAPK11* | mitogen-activated protein kinase 11 |
| *MAPK14* | mitogen-activated protein kinase 14 |
| *MAPKAPK2* | mitogen-activated protein kinase-activated protein kinase 2 |
| *MARCO* | macrophage receptor with collagenous structure |
| *MASP1* | mannan-binding lectin serine peptidase 1 (C4/C2 activating component of Ra-reactive factor) |
| *MASP2* | mannan-binding lectin serine peptidase 2 |
| *MBL2* | mannose-binding lectin (protein C) 2, soluble |
| *MBP* | myelin basic protein |
| *MCL1* | myeloid cell leukemia sequence 1 (BCL2-related) |
| *MIF* | macrophage migration inhibitory factor (glycosylation-inhibiting factor) |
| *MME* | membrane metallo-endopeptidase |
| *MR1* | major histocompatibility complex, class I-related |
| *MRC1* | mannose receptor, C type 1 |
| *MS4A1* | membrane-spanning 4-domains, subfamily A, member 1 |
| *MSR1* | macrophage scavenger receptor 1 |
| *MUC1* | mucin 1, cell surface associated |
| *MX1* | myxovirus (influenza virus) resistance 1, interferon-inducible protein p78 (mouse) |
| *MYD88* | myeloid differentiation primary response gene (88) |
| *NCAM1* | neural cell adhesion molecule 1 |
| *NCF4* | neutrophil cytosolic factor 4, 40kDa |
| *NCR1* | natural cytotoxicity triggering receptor 1 |
| *NFATC1* | nuclear factor of activated T-cells, cytoplasmic, calcineurin-dependent 1 |
| *NFATC2* | nuclear factor of activated T-cells, cytoplasmic, calcineurin-dependent 2 |
| *NFATC3* | nuclear factor of activated T-cells, cytoplasmic, calcineurin-dependent 3 |
| *NFIL3* | nuclear factor, interleukin 3 regulated |
| *NFKB1* | nuclear factor of kappa light polypeptide gene enhancer in B-cells 1 |
| *NFKB2* | nuclear factor of kappa light polypeptide gene enhancer in B-cells 2 (p49/p100) |
| *NFKBIA* | nuclear factor of kappa light polypeptide gene enhancer in B-cells inhibitor, alpha |
| *NFKBIZ* | nuclear factor of kappa light polypeptide gene enhancer in B-cells inhibitor, zeta |
| *NLRP3* | NLR family, pyrin domain containing 3 |
| *NOD1* | nucleotide-binding oligomerization domain containing 1 |
| *NOD2* | nucleotide-binding oligomerization domain containing 2 |
| *NOS2* | nitric oxide synthase 2, inducible |
| *NOTCH1* | notch 1 |
| *NOTCH2* | notch 2 |
| *NT5E* | 5'-nucleotidase, ecto (CD73) |
| *PAX5* | paired box 5 |
| *PDCD1* | programmed cell death 1 |
| *PDCD1LG2* | programmed cell death 1 ligand 2 |
| *PDCD2* | programmed cell death 2 |
| *PDGFB* | platelet-derived growth factor beta polypeptide |
| *PDGFRB* | platelet-derived growth factor receptor, beta polypeptide |
| *PECAM1* | platelet/endothelial cell adhesion molecule |
| *PIGR* | polymeric immunoglobulin receptor |
| *PLA2G2A* | phospholipase A2, group IIA (platelets, synovial fluid) |
| *PLA2G2E* | phospholipase A2, group IIE |
| *PLAU* | plasminogen activator, urokinase |
| *PLAUR* | plasminogen activator, urokinase receptor |
| *PML* | promyelocytic leukemia |
| *POU2F2* | POU class 2 homeobox 2 |
| *PPARG* | peroxisome proliferator-activated receptor gamma |
| *PPBP* | pro-platelet basic protein (chemokine (C-X-C motif) ligand 7) |
| *PRDM1* | PR domain containing 1, with ZNF domain |
| *PRF1* | perforin 1 (pore forming protein) |
| *PRKCD* | protein kinase C, delta |
| *PSMB10* | proteasome (prosome, macropain) subunit, beta type, 10 |
| *PSMB5* | proteasome (prosome, macropain) subunit, beta type, 5 |
| *PSMB7* | proteasome (prosome, macropain) subunit, beta type, 7 |
| *PSMB8* | proteasome (prosome, macropain) subunit, beta type, 8 (large multifunctional peptidase 7) |
| *PSMB9* | proteasome (prosome, macropain) subunit, beta type, 9 (large multifunctional peptidase 2) |
| *PSMC2* | proteasome (prosome, macropain) 26S subunit, ATPase, 2 |
| *PSMD7* | proteasome (prosome, macropain) 26S subunit, non-ATPase, 7 |
| *PTAFR* | platelet-activating factor receptor |
| *PTGER4* | prostaglandin E receptor 4 (subtype EP4) |
| *PTGS2* | prostaglandin-endoperoxide synthase 2 (prostaglandin G/H synthase and cyclooxygenase) |
| *PTK2* | PTK2 protein tyrosine kinase 2 |
| *PTPN2* | protein tyrosine phosphatase, non-receptor type 2 |
| *PTPN22* | protein tyrosine phosphatase, non-receptor type 22 (lymphoid) |
| *PTPN6* | protein tyrosine phosphatase, non-receptor type 6 |
| *PTPRC_all* | protein tyrosine phosphatase, receptor type, C |
| *CD45R0* | protein tyrosine phosphatase, receptor type, C |
| *CD45RA* | protein tyrosine phosphatase, receptor type, C |
| *CD45RB* | protein tyrosine phosphatase, receptor type, C |
| *PYCARD* | PYD and CARD domain containing |
| *RAF1* | v-raf-1 murine leukemia viral oncogene homolog 1 |
| *RAG1* | recombination activating gene 1 |
| *RAG2* | recombination activating gene 2 |
| *RARRES3* | retinoic acid receptor responder (tazarotene induced) 3 |
| *RELA* | v-rel reticuloendotheliosis viral oncogene homolog A (avian) |
| *RELB* | v-rel reticuloendotheliosis viral oncogene homolog B |
| *RORC* | RAR-related orphan receptor C |
| *RUNX1* | runt-related transcription factor 1 |
| *S100A8* | S100 calcium binding protein A8 |
| *S100A9* | S100 calcium binding protein A9 |
| *S1PR1* | sphingosine-1-phosphate receptor 1 |
| *SELE* | selectin E |
| *SELL* | selectin L |
| *SELPLG* | selectin P ligand |
| *SERPING1* | serpin peptidase inhibitor, clade G (C1 inhibitor), member 1 |
| *SH2D1A* | SH2 domain containing 1A |
| *SIGIRR* | single immunoglobulin and toll-interleukin 1 receptor (TIR) domain |
| *SKI* | v-ski sarcoma viral oncogene homolog (avian) |
| *SLAMF1* | signaling lymphocytic activation molecule family member 1 |
| *SLAMF6* | SLAM family member 6 |
| *SLAMF7* | SLAM family member 7 |
| *SLC2A1* | solute carrier family 2 (facilitated glucose transporter), member 1 |
| *SMAD3* | SMAD family member 3 |
| *SMAD5* | SMAD family member 5 |
| *SOCS1* | suppressor of cytokine signaling 1 |
| *SOCS3* | suppressor of cytokine signaling 3 |
| *SPP1* | secreted phosphoprotein 1 |
| *SRC* | v-src sarcoma (Schmidt-Ruppin A-2) viral oncogene homolog (avian) |
| *STAT1* | signal transducer and activator of transcription 1, 91kDa |
| *STAT2* | signal transducer and activator of transcription 2, 113kDa |
| *STAT3* | signal transducer and activator of transcription 3 (acute-phase response factor) |
| *STAT4* | signal transducer and activator of transcription 4 |
| *STAT5A* | signal transducer and activator of transcription 5A |
| *STAT5B* | signal transducer and activator of transcription 5B |
| *STAT6* | signal transducer and activator of transcription 6, interleukin-4 induced |
| *SYK* | spleen tyrosine kinase |
| *TAGAP* | T-cell activation RhoGTPase activating protein |
| *TAL1* | T-cell acute lymphocytic leukemia 1 |
| *TAP1* | transporter 1, ATP-binding cassette, sub-family B (MDR/TAP) |
| *TAP2* | transporter 2, ATP-binding cassette, sub-family B (MDR/TAP) |
| *TAPBP* | TAP binding protein (tapasin) |
| *TBK1* | TANK-binding kinase 1 |
| *TBX21* | T-box 21 |
| *TCF4* | transcription factor 4 |
| *TCF7* | transcription factor 7 (T-cell specific, HMG-box) |
| *TFRC* | transferrin receptor (p90, CD71) |
| *TGFB1* | transforming growth factor, beta 1 |
| *TGFBI* | transforming growth factor, beta-induced, 68kDa |
| *TGFBR1* | transforming growth factor, beta receptor 1 |
| *TGFBR2* | transforming growth factor, beta receptor II (70/80kDa) |
| *THY1* | Thy-1 cell surface antigen |
| *TICAM1* | toll-like receptor adaptor molecule 1 |
| *TIGIT* | T cell immunoreceptor with Ig and ITIM domains |
| *TIRAP* | toll-interleukin 1 receptor (TIR) domain containing adaptor protein |
| *TLR1* | toll-like receptor 1 |
| *TLR2* | toll-like receptor 2 |
| *TLR3* | toll-like receptor 3 |
| *TLR4* | toll-like receptor 4 |
| *TLR5* | toll-like receptor 5 |
| *TLR7* | toll-like receptor 7 |
| *TLR8* | toll-like receptor 8 |
| *TLR9* | toll-like receptor 9 |
| *TMEM173* | transmembrane protein 173 |
| *TNF* | tumor necrosis factor |
| *TNFAIP3* | tumor necrosis factor, alpha-induced protein 3 |
| *TNFAIP6* | tumor necrosis factor, alpha-induced protein 6 |
| *TNFRSF10C* | tumor necrosis factor receptor superfamily, member 10c, decoy without an intracellular domain |
| *TNFRSF11A* | tumor necrosis factor receptor superfamily, member 11a, NFKB activator |
| *TNFRSF13B* | tumor necrosis factor receptor superfamily, member 13B |
| *TNFRSF13C* | tumor necrosis factor receptor superfamily, member 13C |
| *TNFRSF14* | tumor necrosis factor receptor superfamily, member 14 |
| *TNFRSF17* | tumor necrosis factor receptor superfamily, member 17 |
| *TNFRSF1B* | tumor necrosis factor receptor superfamily, member 1B |
| *TNFRSF4* | tumor necrosis factor receptor superfamily, member 4 |
| *TNFRSF8* | tumor necrosis factor receptor superfamily, member 8 |
| *TNFRSF9* | tumor necrosis factor receptor superfamily, member 9 |
| *TNFSF10* | tumor necrosis factor (ligand) superfamily, member 10 |
| *TNFSF11* | tumor necrosis factor (ligand) superfamily, member 11 |
| *TNFSF12* | tumor necrosis factor (ligand) superfamily, member 12 |
| *TNFSF13B* | tumor necrosis factor (ligand) superfamily, member 13b |
| *TNFSF15* | tumor necrosis factor (ligand) superfamily, member 15 |
| *TNFSF4* | tumor necrosis factor (ligand) superfamily, member 4 |
| *TNFSF8* | tumor necrosis factor (ligand) superfamily, member 8 |
| *TOLLIP* | toll interacting protein |
| *TP53* | tumor protein p53 |
| *TRAF1* | TNF receptor-associated factor 1 |
| *TRAF2* | TNF receptor-associated factor 2 |
| *TRAF3* | TNF receptor-associated factor 3 |
| *TRAF4* | TNF receptor-associated factor 4 |
| *TRAF5* | TNF receptor-associated factor 5 |
| *TRAF6* | TNF receptor-associated factor 6 |
| *TYK2* | tyrosine kinase 2 |
| *UBE2L3* | ubiquitin-conjugating enzyme E2L 3 |
| *VCAM1* | vascular cell adhesion molecule 1 |
| *VTN* | vitronectin |
| *XBP1* | X-box binding protein 1 |
| *XCL1* | chemokine (C motif) ligand 1 |
| *XCR1* | chemokine (C motif) receptor 1 |
| *ZAP70* | zeta-chain (TCR) associated protein kinase 70kDa |
| *ZBTB16* | zinc finger and BTB domain containing 16 |
| *ZEB1* | zinc finger E-box binding homeobox 1 |

| **Supplementary Table 1B**. Genes included in each pathway analysis | |
| --- | --- |
| **Pathways** | **Genes included in the analysis** |
| Cell Adhesion | *CD2, CD4, CD6, CD8A, CD8B, CD22, CD28, CD34, CD40, CD40LG, CD45RA, CD45RB, CD45R0, CD58, CD80, CD86, CD99, CDH5, CD274, CD276, CTLA4-TM, CTLA4_all, HLA-A, HLA-B, HLA-C, HLA-DMA, HLA-DMB, HLA-DOB, HLA-DPA1, HLA-DPB1, HLA-DQA1, HLA-DQB1, HLA-DRA, HLA-DRB1, HLA-DRB3, ICAM1, ICAM2, ICAM3, ICOS, ICOSLG, ITGA4, ITGA6, ITGAE, ITGAL, ITGAM, ITGAX, ITGB1, ITGB2, NCAM1, PDCD1, PDCD1LG2, PECAM1, PTPRC, SELE, SELL, SELPLG, TGFBI, TIGIT, VCAM1* |
| MHC Class II Antigen Presentation | *CD74, CTSC, CTSS, HLA-DMA, HLA-DMB, HLA-DOB, HLA-DPA1, HLA-DPB1, HLA-DQA1, HLA-DQB1, HLA-DRA, HLA-DRB1, HLA-DRB3, LAG3* |
| Type II Interferon Signaling | *B2M, CD44, CIITA, FCGR1A/B, GBP1, GBP5, HLA-A, HLA-B, HLA-C, HLA-DPA1, HLA-DPB1, HLA-DQA1, HLA-DQB1, HLA-DRA, HLA-DRB1, HLA-DRB3, ICAM1, IFNG, IFNGR1, IRF1, IRF3, IRF4, IRF5, IRF7, IRF8, JAK1, JAK2, NCAM1, PML, PRKCD, PTAFR, PTPN6, SOCS1, SOCS3, STAT1, VCAM1* |
| T Cell Receptor Signaling | *BCL10, CD247, CD28, CD3D, CD3E, CD40LG, CD45RA, CD45RB, CD45R0, CD4, CD8A, CD8B, CHUK, CSF2, CTLA4-TM, CTLA4_*all*, FYN, HLA-DPA1, HLA-DPB1, HLA-DQA1, HLA-DQB1, HLA-DRA, HLA-DRB1, HLA-DRB3, HRAS, ICOS, IFNG, IL10, IL2, IL4, IKBKB, IKBKG, LCK, LCP2, MALT1, MAPK1, MAPK11, MAPK14, NFATC1, NFATC2, NFATC3, NFKB1, NFKBIA, PDCD1, PSMB10, PSMB5, PSMB7, PSMB8, PSMB9, PSMC2, PSMD7, PTPN22, PTPN6, PTPRC_*all*, RAF1, RELA, TNF, TRAF6, ZAP70* |
| Phagocytosis and Degradation | *C1R, C3, CD14, CD163, CD164, CD209, CD36, CLEC7A, CTSC, CTSS, CTSG, CYBB, FCGR1A/B, FCGR2A, FCGR2A/C, FCGR2B, FCGR3A/B, HLA-A, HLA-B, HLA-C, HLA-DMA, HLA-DMB, HLA-DOB, HLA-DPA1, HLA-DPB1, HLA-DQA1, HLA-DQB1, HLA-DRA, HLA-DRB1, HLA-DRB3, IGF2R, ITGA5, ITGAM, ITGB1, ITGB2, LAMP3, LITAF, MARCO, MBL2, MRC1, MSR1, NCF4, TAP1, TAP2, TFRC, TLR2, TLR4* |
| Adaptive Immune System | *ATG7, B2M, BCL10, BCAP31, BLNK, BTK, BTLA, C3, CD1A, CD1D, CD14, CD19, CD22, CD28, CD36, CD3D, CD3E, CD4, CD40LG, CD45RA, CD45RB, CD45R0, CD74, CD79A, CD79B, CD80, CD81, CD86, CD8A, CD8B, CD96, CD99, CD160, CHUK, CTLA4-TM, CTLA4_all, CTSC, CYBB, FYN, FCGR1A/B, FCGR2B, HLA-A, HLA-B, HLA-C, HLA-DMB, HLA-DOB, HLA-DPA1, HLA-DPB1, HLA-DQA1, HLA-DQB1, HLA-DRA, HLA-DRB1, HLA-DRB3, HRAS, ICAM1, ICAM2, ICAM3, ICAM4, ICAM5, ICOSLG, IFITM1, IKBKB, IKBKG, ITGA4, ITGAL, ITGB1, ITGB2, KIR3DL1, KIR3DL2, KIR3DL3, KIR_Activating_Subgroup_2, KIR_*Inhibiting_Subgroup_1*,* KIR_Inhibiting_Subgroup_2*, KLRB1, KLRC1, KLRD1, KLRF1, KLRG1, LAG3, LAIR1, LCP2, LCK, LILRA1, LILRA2, LILRA3, LILRA4, LILRA5, LILRA6, LILRB1, LILRB3, LILRB4, LILRB5, LY96, MALT1, MRC1, MYD88, NCR1, NCF4, NFATC1, NFATC2, NFATC3, NFKB1, NFKBIA, PSMB5, PSMB7, PSMB8, PSMB9, PSMB10, PSMC2, PSMD7, PDCD1, PDCD1LG2, PTPN6, PTPN22, PTPRC_*all*, RAF1, RELA, SELL, SH2D1A, SLAMF6, SLAMF7, SOCS1, SOCS3, SYK, TAP1, TAP2, TAPBP, TIRAP, TLR1, TLR2, TLR4, TNFRSF14, TRAF6, UBE2L3, VCAM1, ZAP70, ZBTB16* |
| Type I Interferon Signaling | *BST2, EGR1, HLA-A, HLA-B, HLA-C, IFI35, IFIT2, IFITM1, IFNA2, IFNAR1, IFNAR2, IRF1, IRF3, IRF4, IRF5, IRF7, IRF8, JAK1, MX1, PSMB8, PTPN6, SOCS1, SOCS3, STAT1, STAT2, TYK2* |
| MHC Class I Antigen Presentation | *ATG7, B2M, BCAP31, BTK, CD14, CD36, CHUK, CYBB, FCGR1A/B, HLA-A, HLA-B, HLA-C, IKBKB, IKBKG, LILRA6, LY96, MRC1, MR1, MYD88, NCF4, PSMB10, PSMB5, PSMB7, PSMB8, PSMB9, PSMC2, PSMD7, SOCS1, SOCS3, TAP1, TAP2, TAPBP, TIRAP, TLR1, TLR2, TLR4, UBE2L3, ZBTB16* |
| Lymphocyte Activation | ABL1, ADA, AHR, AICDA, ATG5, ATM, B2M, BATF, BAX, BCL10, BCL2, BCL3, BCL6, BID, BLNK, BTK, BTLA, BST1, BST2, CCL19, CCL2, CCL5, CD1D, CD209, CD244, CD24, CD247, CD27, CD274, CD28, CD2, CD3D, CD3E, CD40, CD40LG, CD44, CD45RA, CD45RB, CD45R0, CD46, CD48, CD5, CD55, CD59, CD6, CD70, CD74, CD79A, CD79B, CD80, CD81, CD83, CD86, CD8A, CD8B, CDKN1A, CEACAM1, CEACAM6, CEBPB, CR2, CSF2, CTLA4-TM, CTLA4_all, CTNNB1, EBI3, EGR1, EOMES, FADD, FAS, FCER1G, FCGR3A/B, FN1, FOXP3, FYN, GATA3, GPR183, GZMA, GZMB, GZMK, HAVCR2, HFE, HLA-A, HLA-B, HLA-C, HLA-DMB, HLA-DOB, HLA-DPA1, HLA-DPB1, HLA-DQA1, HLA-DQB1, HLA-DRA, HLA-DRB1, HLA-DRB3, HRAS, ICAM1, ICAM2, ICOS, ICOSLG, IDO1, IFNA2, IFNG, IFNAR1, IFNAR2, IFNGR1, IKZF1, IKZF3, IL10, IL12A, IL12B, IL12RB1, IL13, IL15, IL18, IL18R1, IL1B, IL21, IL21R, IL23A, IL23R, IL27, IL28A, IL28A/B, IL2, IL2RA, IL2RB, IL2RG, IL4, IL4R, IL6, IL6ST, IL7, IL7R, IL8, IRF1, IRF4, JAK3, KIR3DL1, KIR3DL2, KIR3DL3, KIR_*Activating_Subgroup_1*, KIR_*Activating_Subgroup_2*, KIR_*Inhibiting_Subgroup_1*, KIR_*Inhibiting_Subgroup_2,* KIT, KLRC1, KLRC2, KLRC3, KLRC4, KLRD1, KLRG1, KLRG2, KLRK1, LAG3, LCK, LEF1, LGALS3, LILRB1, LILRB2, MALT1, MAPK1, MYD88, NFATC1, NFATC2, NFKB2, NOTCH2, NLRP3, NOD2, POU2F2, PRDM1, PRF1, PRKCD, PSMA10, PSMB10, PSMB5, PSMC2, PSMD7, PTGER4, PTPN22, PTPN6, PYCARD, RAF1, RAG1, RAG2, RELB, RORC, SERPING1, SH2D1A, SLAMF1, SLAMF6, SLAMF7, SMAD3, SOCS1, SRC, STAT5B, STAT6, SYK, TBK1, TCF7, TGFBR2, TGFB1, THY1, TICAM1, TIGIT, TIRAP, TLR4, TNFAIP3, TNFRSF10C, TNFRSF13B, TNFRSF13C, TNFRSF14, TNFRSF1B, TNFRSF4, TNFRSF9, TNFSF10, TNFSF11, TNFSF13B, TNFSF4, TNFSF8, TP53, TRAF2, TRAF6, VCAM1, XBP1, XCL1, ZAP70, ZBTB16, ZEB1 |
| Chemokine Signaling | *CCBP2, CCL11, CCL13, CCL16, CCL18, CCL19, CCL2, CCL20, CCL22, CCL23, CCL24, CCL26, CCL3, CCL4, CCL5, CCL7, CCL8, CCR1, CCR10, CCR2, CCR5, CCR6, CCR7, CCR8, CCRL1, CCRL2, CHUK, CX3CL1, CX3CR1, CXCL1, CXCL10, CXCL11, CXCL12, CXCL13, CXCL2, CXCL9, CXCR1, CXCR2, CXCR3, CXCR4, CXCR6, HRAS, IKBKB, IKBKG, IL8, JAK2, JAK3, MAPK1, NFKB1, NFKBIA, PPBP, PRKCD, PTK2, RAF1, RELA, SRC, STAT1, STAT2, STAT3, STAT5B, XCL1, XCR1* |
| Hemostasis | *ABL1, APP, C1QBP, CD2, CD244, CD36, CD44, CD48, CD58, CD74, CD9, CD99, CEACAM1, CEACAM6, CEACAM8, CLU, CSF2, CSF2RB, EDNRB, FCER1G, FCGRT, FN1, FYN, GATA3, GP1BB, HAMP, HRAS, IFNA2, IL2, IL2RA, IL2RB, IL2RG, IL3, IRF1, IRF3, IRF7, ITGA2B, ITGA4, ITGA5, ITGA6, ITGAL, ITGAM, ITGAX, ITGB1, ITGB2, JAK1, JAK2, JAK3, LCK, LCP2, MAPK1, MAPK14, MIF, NOS2, PDGFB, PECAM1, PLAU, PLAUR, PPBP, PRKCD, PTK2, RAF1, SELPLG, SELE, SELL, SERPING1, SYK, TGFB1, TP53* |
| NF-kB Signaling | *ATM, BCL10, BCL2, BLNK, BTK, CCL13, CCL19, CCL4, CD14, CD40, CD40LG, CXCL12, CXCL2, ICAM1, IKBKB, IKBKG, IKBKAP, IL1B, IL1R1, IL8, IRAK1, IRAK4, LCK, LTBR, LTA, LY96, MALT1, MYD88, NFKB1, NFKB2, NFKBIA, PLAU, PSMB10, PSMB5, PSMB7, PSMB8, PSMB9, PSMC2, PSMD7, PTGS2, RELA, RELB, SYK, TAGAP, TICAM1, TIRAP, TLR4, TNF, TNFAIP3, TNFRSF11A, TNFRSF13C, TNFSF11, TNFSF13B, TRAF1, TRAF2, TRAF3, TRAF4, TRAF5, TRAF6, ZAP70* |
| Cytokine Signaling | *APP, B2M, BATF, BCL2, BCL6, BLNK, BST2, CASP1, CASP3, CCL11, CCL13, CCL16, CCL18, CCL19, CCL2, CCL20, CCL22, CCL23, CCL26, CCL3, CCL4, CCL5, CCL7, CCL8, CCR1, CCR10, CCR2, CCR5, CCR6, CCR7, CCR8, CD19, CD27, CD36, CD4, CD40, CD40LG, CD44, CD70, CD80, CD86, CDKN1A, CIITA, CISH, CSF1, CSF1R, CSF2, CSF2RB, CSF3R, CX3CL1, CX3CR1, CXCL1, CXCL10, CXCL11, CXCL12, CXCL13, CXCL2, CXCL9, CXCR1, CXCR2, CXCR3, CXCR4, CXCR6, DUSP4, EBI3, EGR1, FAS, FCGR1A/B, FN1, FYN, GATA3, GBP1, GBP5, HAVCR2, HLA-A, HLA-B, HLA-C, HLA-DPA1, HLA-DPB1, HLA-DQA1, HLA-DQB1, HLA-DRA, HLA-DRB1, HLA-DRB3, HRAS, ICAM1, IFI35, IFIT2, IFITM1, IFNA2, IFNG, IFNAR1, IFNAR2, IFNGR1, IL1A, IL1B, IL10, IL10RA, IL11RA, IL12A, IL12B, IL12RB1, IL13, IL13RA1, IL15, IL16, IL17B, IL17F, IL18, IL18R1, IL18RAP, IL19, IL1R1, IL1R2, IL1RAP, IL1RL1, IL1RL2, IL1RN, IL2, IL20, IL21, IL21R, IL22, IL23A, IL23R, IL26, IL27, IL28A, IL28A/B, IL2RA, IL2RB, IL2RG, IL32, IL3, IL4, IL4R, IL6, IL6R, IL6ST, IL7, IL7R, IL8, IRF1, IRF3, IRF4, IRF5, IRF7, IRF8, IRAK1, IRAK2, IRAK3, IRAK4, ITGAM, ITGAX, ITGB1, ITGB2, JAK1, JAK2, JAK3, KIT, LCK, LIF, LTBR, MAPK1, MAPK11, MAPK14, MAPKAPK2, MCL1, MIF, MUC1, MX1, NCAM1, NFKB1, NFKB2, NFKBIA, NOS2, NOD1, NOD2, PDGFB, PDGFRB, PML, PSMB10, PSMB5, PSMB7, PSMB8, PSMB9, PSMC2, PSMD7, PTAFR, PTGS2, PTPN2, PTPN6, PPBP, PRKCD, RAG1, RAG2, RAF1, RELA, RELB, RORC, S1PR1, SIGIRR, SMAD3, SOCS1, SOCS3, STAT1, STAT2, STAT3, STAT4, STAT5A, STAT5B, STAT6, TBK1, TGFB1, TGFBR1, TGFBR2, TLR4, TNF, TNFAIP3, TNFRSF10C, TNFRSF11A, TNFRSF13B, TNFRSF13C, TNFRSF14, TNFRSF17, TNFRSF1B, TNFRSF4, TNFRSF8, TNFSF10, TNFSF11, TNFSF12, TNFSF13B, TNFSF15, TNFSF4, TNFSF8, TOLLIP, TP53, TRAF2, TRAF3, TRAF6, TYK2, VCAM1, XCL1, XCR1, ZEB1* |
| B cell Receptor Signaling | *BCL10, BLNK, BTK, CD19, CD22, CD79A, CD79B, CD81, CHUK, CR2, FCGR2B, FYN, HRAS, IFITM1, IKBKB, IKBKG, LILRB3, MALT1, MAPK1, NFATC1, NFATC2, NFATC3, NFKB1, NFKBIA, PSMB5, PSMB7, PSMB8, PSMB9, PSMB10, PSMC2, PSMD7, PTPN6, RAF1, RELA, SYK* |
| Host-pathogen Interaction | *ABL1, APP, ARG1, ARG2, ATG5, ATM, BAX, BCL10, BCL2, BCAP31, BID, CAMP, C1QA, C1QB, C1QBP, C1R, C1S, C2, C3, C4A/B, C5, C8B, C8G, C9, CARD9, CASP1, CASP10, CASP3, CASP8, CCL2, CCL3, CCL4, CCL5, CD1D, CD14, CD19, CD209, CD247, CD28, CD3D, CD3E, CD3EAP, CD36, CD40, CD40LG, CD44, CD46, CD58, CD74, CD81, CDKN1A, CEACAM1, CEBPB, CFH, CFB, CFD, CFI, CFP, CHUK, CIITA, CLEC4E, CLEC7A, CR1, CR2, CSF2, CYBB, CTSG, CTNNB1, CTSS, CXCL1, CXCL10, CXCL2, CXCR1, CXCR2, CXCR3, CXCR4, CXCR6, DUSP4, EGR1, EGR2, ENTPD1, ETS1, FADD, FAS, FCER1G, FCGR1A/B, FCGR2A, FCGR2A/C, FCGR2B, FCGR3A/B, FN1, FYN, HAVCR2, HLA-A, HLA-B, HLA-C, HLA-DMA, HLA-DMB, HLA-DOB, HLA-DPA1, HLA-DPB1, HLA-DQA1, HLA-DQB1, HLA-DRA, HLA-DRB1, HLA-DRB3, HRAS, ICAM1, IDO1, IFIHI, IFITM1, IFNA2, IFNG, IFNAR1, IFNAR2, IFNGR1, IL10, IL10RA, IL12A, IL12B, IL13, IL15, IL18, IL1A, IL1B, IL1R1, IL1R2, IL2, IL20, IL23A, IL2RA, IL2RB, IL2RG, IL28A, IL3, IL4, IL46, IL6, IL8, IRF1, IRF3, IRF7, IRF8, IRAK1, IRAK2, IRAK4, ITGA2B, ITGA4, ITGA5, ITGA6, ITGAL, ITGAM, ITGAX, ITGB1, ITGB2, JAK1, JAK2, JAK3, KIT, KLRB1, KLRK1, LCK, LTBR, LY96, MALT1, MAPK1, MAPK11, MAPK14, MASP1, MASP2, MBL2, MRC1, MX1, MYD88, NCAM1, NCF4, NFATC1, NFATC2, NFATC3, NFKB1, NFKB2, NFKBIA, NLRP3, NOD1, NOD2, NOS2, PML, PDGFB, PDGFRB, PECAM1, PPBP, PRKCD, PSAF1, PSMD7, PSMC2, PSMB10, PSMB5, PSMB7, PSMB8, PSMB9, PTAFR, PTGER4, PTGS2, PTPN6, PYCARD, RAF1, RELA, RELB, SERPING1, SH2D1A, SIGIRR, SLC2A1, SLAMF1, SMAD3, SOCS1, SOCS3, SPP1, SRC, STAT1, STAT2, STAT3, STAT4, STAT5A, STAT5B, STAT6, SYK, TAP1, TAP2, TBK1, TCF7, TGFBR1, TGFBR2, TGFB1, TICAM1, TIRAP, TLR1, TLR2, TLR3, TLR4, TLR5, TLR7, TLR9, TMEM173, TNF, TNFAIP3, TNFRSF10C, TNFRSF13C, TNFRSF14, TNFRSF1B, TNFRSF4, TNFSF10, TRAF1, TRAF2, TRAF3, TRAF5, TRAF6, TYK2, VCAM1, VTN, XBP1* |
| Treg Differentiation | FOXP3, IL10, IL10RA, JAK1, SMAD3, STAT3, TGFB1, TGFBR1, TGFBR2, TYK2 |
| Lymphocyte Trafficking | *ARHGDIB, CD99, CDH5, CTNNB1, CXCL12, CXCR4, CYBB, ICAM1, ITGA4, ITGAL, ITGAM, ITGB1, ITGB2, MAPK11, MAPK14, MBP, NCF4, PECAM1, PTK2, THY1, VCAM1* |
| Innate Immune System | *ABL1, APP, ARG1, ATG12, ATG5, ATG7, B2M, BCL10, BCL2, BST1, BST2, BTK, C1QA, C1QB, C1R, C1S, C2, C3, C4A/B, C5, C6, C7, C8B, C8G, C9, CAMP, CARD9, CASP1, CASP10, CASP2, CASP8, CD14, CD19, CD209, CD36, CD4, CD44, CD45RA, CD45RB, CD45R0, CD46, CD53, CD55, CD58, CD59, CD81, CD97, CEACAM1, CEACAM6, CEACAM8, CFH, CFB, CFD, CFI, CFP, CHUK, CLEC4A, CLEC4E, CLEC5A, CLEC6A, CLEC7A, CLU, CR1, CR2, CTNNB1, CTSC, CTSG, CYBB, CXCL1, CXCR1, CXCR2, DEFB103A, DEFB103B, DEFB1, DEFB4A, DUSP4, FCER1A, FCER1G, FCGR1A/B, FCGR2A, FCGR3A/B, FN1, FYN, GPI, HRAS, ICAM2, ICAM3, IFI16, IFIH1, IFNA2, IKBKB, IKBKE, IKBKG, IRF1, IRF3, IRF7, IRAK1, IRAK2, IRAK3, IRAK4, ITGAL, ITGAM, ITGAX, ITGB2, ITLN1, ITLN2, JAK1, JAK2, JAK3, KIR_*Activating_Subgroup_1*, KIR_*Activating_Subgroup_2*, KLRD1, LAIR1, LCK, LGALS3, LILRA3, LTF, LY96, MALT1, MAPK1, MAPK11, MAPK14, MAPKAPK2, MASP1, MBL2, MIF, MME, MUC1, MYD88, NCF4, NFATC1, NFATC2, NFATC3, NFKB1, NFKB2, NFKBIA, NLRP3, NOD1, NOD2, NOS2, PECAM1, PIGR, PLA2G2A, PLAU, PLAUR, PPBP, PRKCD, PSMA5, PSMB10, PSMB5, PSMB7, PSMB8, PSMB9, PSMC2, PSMD7, PTAFR, PTGS2, PTPN6, PYCARD, RAF1, RELA, RELB, SELL, SELE, SERPING1, SIGIRR, SLC2A1, SLAMF1, SOCS1, STAT6, SYK, TIRAP, TICAM1, TLR1, TLR2, TLR3, TLR4, TLR5, TLR7, TLR8, TLR9, TMEM173, TNFAIP3, TNFAIP6, TNFRSF1B, TNFRSF10C, TNFRSF14, TRAF2, TRAF3, TRAF6, VCAM1, VTN* |
| TNF Family Signaling | *BCL3, CCL2, CCL20, CCL5, CEBPB, CASP10, CASP3, CASP8, CHUK, CSF1, CSF2, CX3CL1, CXCL1, CXCL10, CXCL2, FADD, FAS, ICAM1, IKBKB, IKBKG, IL15, IL18R1, IL1B, IL6, LIF, LTA, MAP4K1, MAP4K2, MAP4K4, MAPK1, MAPK11, MAPK14, NFKB1, NFKBIA, NOD2, PTGS2, RELA, SELE, SOCS3, TNF, TNFAIP3, TNFRSF1B, TNFSF10, TRAF1, TRAF2, TRAF3, TRAF4, TRAF5, VCAM1* |
| Transcriptional Regulation | *AHR, BATF, BATF3, BCL3, CEBPB, EGR1, EGR2, EOMES, ETS1, FOXP3, GATA3, GFI1, IKZF1, IKZF2, IKZF3, ILF3, IRF1, IRF3, IRF4, IRF5, IRF7, IRF8, LEF1, MAF, NFATC1, NFATC2, NFATC3, NFKB1, NFKB2, NFKBIA, NFKBIZ, NFIL3, PAX5, POU2F2, PPARG, RELA, RELB, RORC, RUNX1, STAT1, STAT2, STAT3, STAT4, STAT5A, STAT5B, STAT6, TAL1, TCF4, TCF7, TBX21, TP53, XBP1, ZEB1* |
| Th2 Differentiation | *GATA3, IL13, IL2, IL2RA, IL2RB, IL2RG, IL4, IL4R, IL6, JAK3, MAF, NOTCH1, NOTCH2, STAT5A, STAT5B, STAT6* |
| Oxidative Stress | *ABL1, ADA, APP, ARG1, BCL2, CASP3, CCL19, CCL5, CCR7, CD36, CHUK, CYBB, ETS1, FAS, FN1, IL18RAP, IL1B, IL1R1, IL6, JAK2, MBL2, MCL1, NFKB1, PDGFRB, PML, PRKCD, PSMB5, RELA, SRC, STAT6, TNF, TNFAIP3, TP53, XBP1* |
| Apoptosis | *ARHGDIB, ATM, BAX, BCL2, BCL2L11, BCAP31, BID, CASP2, CASP3, CASP8, CASP10, CD14, CD82, CRADD, CSF2RB, CTSC, CTSS, CUL9, FADD, FAS, GZMA, GZMB, GZMK, HRAS, IKBKB, IKBKG, IL3, LY96, MAPK1, MCL1, NFKB1, NFKBIA, PDCD2, PRF1, PRKCD, PSMB5, PSMB7, PSMB8, PSMB9, PSMB10, PSMC2, PSMD7, PTK2, RAF1, RELA, TICAM1, TNF, TNFRSF10C, TNFSF10, TP53, TRAF1, TRAF2, TLR4* |
| Complement System | *C1QA, C1QB, C1QBP, C1R, C1S, C2, C3, C4A/B, C5, C6, C7, C8B, C8G, C9, CD19, CD46, CD55, CD59, CD81, CFH, CFB, CFD, CFI, CFP, CLU, CR1, CR2, ITGAM, ITGAX, ITGB2, MASP1, MASP2, MBL2, PLAU, PLAUR, SERPING1, VTN* |
| TLR Signaling | *APP, BTK, CCL3, CCL4, CCL5, CD14, CD36, CD40, CD80, CD86, CHUK, CASP8, CTSS, CXCL10, CXCL11, CXCL9, DUSP4, FADD, IFNA2, IFNAR1, IFNAR2, IL12A, IL12B, IL1B, IL6, IL8, IRF3, IRF5, IRF7, IKBKB, IKBKE, IKBKG, LY96, MAPK1, MAPK11, MAPK14, MAPKAPK2, MYD88, NFKB1, NFKB2, NFKBIA, NOD1, NOD2, RELA, S100A8, S100A9, SIGIRR, SOCS1, SPP1, STAT1, TBK1, TICAM1, TIRAP, TLR1, TLR2, TLR3, TLR4, TLR5, TLR7, TLR8, TLR9, TNF, TOLLIP, TRAF3, TRAF6* |
| Th1 Differentiation | *IFNG, IFNGR1, IL12A, IL12B, IL12RB1, IL2, JAK1, JAK2, NFKB1, RELA, STAT1, STAT4, TBX21, TYK2* |
| NLR signaling | *APP, ATG12, ATG16L1, ATG5, BCL2, CAMP, CARD9, CASP1, CASP2, CASP8, CCL2, CCL5, CHUK, CXCL1, CXCL2, CYBB, FADD, GBP1, GBP5, IFI16, IFNA2, IFNAR1, IFNAR2, IKBKB, IKBKE, IKBKG, IL18, IL1B, IL6, IL8, IRF3, IRF7, IRAK1, IRAK2, IRAK4, JAK1, MAPK1, MAPK11, MAPK14, MYD88, NFKB1, NFKB2, NFKBIA, NLRP3, NOD1, NOD2, PRKCD, PYCARD, RELA, STAT1, STAT2, TBK1, TICAM1, TLR4, TMEM173, TNF, TNFAIP3, TRAF2, TRAF3, TRAF5, TRAF6, TYK2* |
| Th17 Differentiation | *AHR, IL12B, IL12RB1, IL17F, IL1B, IL1R1, IL1RAP, IL21, IL21R, IL22, IL23A, IL23R, IL2RG, IL6, IL6R, IL6ST, IRF4, JAK1, JAK2, JAK3, MAPK11, MAPK14, RORC, SMAD3, STAT3, TGFB1, TGFBR1, TGFBR2, TNF, TYK2* |
| Autophagy | *ATG10, ATG12, ATG16L1, ATG5, ATG7, BCL2, HRAS, MAPK1, PRKCD, RAF1, TRAF6* |
| TGF-b Signaling | *IFNG, MAPK1, SKI, SMAD3, SMAD5, TGFB1, TGFBR1, TGFBR2, TNF* |
| Immunometabolism | *ABCB1, ADA, AHR, ARG1, ARG2, B3GAT1, BST1, CD36, CD44, CMKLR1, ENTPD1, FKBP5, GPI, IDO1, KCNJ2, LTB4R, LTB4R2, MAPKAPK2, NT5E, PLA2G2A, PLA2G2E, PPARγ, PSMB5, PSMB7, PSMB8, PSMB9, PSMB10, PSMC2, PSMD7, PTGS2, RARRES3, SLC2A1* |
| Inflammasomes | *APP, BCL2, CASP1, NFKB1, NFKB2, NLRP3, PYCARD, RELA* |

| **Supplementary Table 1C.** Differentially expressed genes between brisk and non-brisk TIL groups. | | | | | |
| --- | --- | --- | --- | --- | --- |
| **Genes** | **Log_2_ Fold Change** | **Upregulated group** | **p-value** | **Adjusted p-value** | **Statistical method** |
| *HLA-C* | 1.650 | Brisk | < 0.001 | 0.005 | Log-linear negative binomial model |
| *SELL* | 2.000 | Brisk | < 0.001 | 0.036 | Log-linear negative binomial model |
| *TLR1* | 1.120 | Brisk | < 0.001 | 0.046 | Log-linear negative binomial model |
| *ZAP70* | 1.310 | Brisk | < 0.001 | 0.046 | Log-linear negative binomial model |
| *CCND3* | 0.853 | Brisk | 0.001 | 0.072 | Log-linear negative binomial model |
| *CD45R0* | 1.330 | Brisk | 0.001 | 0.072 | Log-linear negative binomial model |
| *PTPRC* | 1.030 | Brisk | 0.001 | 0.072 | Log-linear negative binomial model |
| *HLA-A* | 1.060 | Brisk | 0.001 | 0.072 | Log-linear negative binomial model |
| *HLA-DPA1* | 1.170 | Brisk | 0.001 | 0.072 | Log-linear negative binomial model |
| *ITGAL* | 1.350 | Brisk | 0.001 | 0.072 | Log-linear negative binomial model |
| *TAGAP* | 1.350 | Brisk | 0.001 | 0.074 | Log-linear negative binomial model |
| *PTPN6* | 1.050 | Brisk | 0.002 | 0.077 | Log-linear negative binomial model |
| *IL1B* | 2.710 | Brisk | 0.002 | 0.097 | Log-linear negative binomial model |
| *CD48* | 1.370 | Brisk | 0.002 | 0.097 | Log-linear negative binomial model |
| *UBE2L3* | 0.649 | Brisk | 0.003 | 0.097 | Log-linear negative binomial model |
| *IL7R* | 1.640 | Brisk | 0.003 | 0.097 | Log-linear negative binomial model |
| *TCF7* | 1.440 | Brisk | 0.003 | 0.097 | Log-linear negative binomial model |
| *TAP1* | 1.180 | Brisk | 0.003 | 0.097 | Log-linear negative binomial model |
| *CD3D* | 1.540 | Brisk | 0.003 | 0.097 | Mixture negative binomial model |
| *LITAF* | 0.738 | Brisk | 0.004 | 0.105 | Log-linear negative binomial model |
| *HLA-B* | 1.020 | Brisk | 0.004 | 0.110 | Log-linear negative binomial model |
| *FCGR3A/B* | 1.010 | Brisk | 0.004 | 0.112 | Log-linear negative binomial model |
| *IRF8* | 0.989 | Brisk | 0.005 | 0.124 | Log-linear negative binomial model |
| *PSMB9* | 0.931 | Brisk | 0.005 | 0.124 | Log-linear negative binomial model |
| *HLA-DPB1* | 0.992 | Brisk | 0.006 | 0.124 | Log-linear negative binomial model |
| *CCL20* | 1.710 | Brisk | 0.006 | 0.124 | Mixture negative binomial model |
| *CXCR2* | 1.620 | Brisk | 0.006 | 0.133 | Mixture negative binomial model |
| *MS4A1* | 1.820 | Brisk | 0.008 | 0.158 | Mixture negative binomial model |
| *CD53* | 1.090 | Brisk | 0.009 | 0.170 | Log-linear negative binomial model |
| *CSF3R* | 1.480 | Brisk | 0.010 | 0.174 | Mixture negative binomial model |
| *CCL19* | 1.500 | Brisk | 0.010 | 0.174 | Log-linear negative binomial model |
| *CD36* | -1.450 | Non-brisk | 0.011 | 0.174 | Log-linear negative binomial model |
| *CD74* | 0.771 | Brisk | 0.012 | 0.192 | Log-linear negative binomial model |
| *PSMB10* | 0.614 | Brisk | 0.014 | 0.200 | Log-linear negative binomial model |
| *NFATC2* | -1.030 | Non-brisk | 0.014 | 0.211 | Log-linear negative binomial model |
| *IL1R2* | 1.240 | Brisk | 0.014 | 0.211 | Mixture negative binomial model |
| *GPI* | -0.587 | Non-brisk | 0.015 | 0.211 | Log-linear negative binomial model |
| *IL10RA* | 0.726 | Brisk | 0.015 | 0.211 | Log-linear negative binomial model |
| *HLA-DMB* | 0.851 | Brisk | 0.015 | 0.211 | Log-linear negative binomial model |
| *HLA-DRA* | 0.915 | Brisk | 0.016 | 0.211 | Log-linear negative binomial model |
| *NCF4* | 0.774 | Brisk | 0.016 | 0.211 | Log-linear negative binomial model |
| *IL1RN* | 1.320 | Brisk | 0.016 | 0.211 | Log-linear negative binomial model |
| *CIITA* | 1.270 | Brisk | 0.018 | 0.211 | Mixture negative binomial model |
| *IRF1* | 0.763 | Brisk | 0.019 | 0.228 | Log-linear negative binomial model |
| *BCL6* | 0.666 | Brisk | 0.020 | 0.228 | Log-linear negative binomial model |
| *SERPING1* | 0.814 | Brisk | 0.020 | 0.228 | Log-linear negative binomial model |
| *LCP2* | 0.719 | Brisk | 0.022 | 0.228 | Log-linear negative binomial model |
| *TNFRSF17* | 1.340 | Brisk | 0.023 | 0.228 | Mixture negative binomial model |
| *CD79A* | 1.570 | Brisk | 0.024 | 0.228 | Mixture negative binomial model |
| *TNFRSF1B* | 0.600 | Brisk | 0.024 | 0.252 | Log-linear negative binomial model |
| *CD19* | 1.370 | Brisk | 0.025 | 0.254 | Mixture negative binomial model |
| *ITGB2* | 0.698 | Brisk | 0.026 | 0.255 | Log-linear negative binomial model |
| *SLAMF7* | 1.100 | Brisk | 0.027 | 0.255 | Log-linear negative binomial model |
| *CYBB* | 0.732 | Brisk | 0.028 | 0.260 | Log-linear negative binomial model |
| *C7* | 1.610 | Brisk | 0.030 | 0.263 | Mixture negative binomial model |
| *PRF1* | 1.140 | Brisk | 0.030 | 0.268 | Mixture negative binomial model |
| *CXCR4* | 0.733 | Brisk | 0.030 | 0.275 | Log-linear negative binomial model |
| *CSF2RB* | 0.809 | Brisk | 0.037 | 0.285 | Log-linear negative binomial model |
| *CD70* | 1.060 | Brisk | 0.037 | 0.285 | Mixture negative binomial model |
| *MAP4K1* | 0.657 | Brisk | 0.038 | 0.285 | Log-linear negative binomial model |
| *IL8* | 1.640 | Brisk | 0.043 | 0.340 | Log-linear negative binomial model |
| *RARRES3* | 1.080 | Brisk | 0.044 | 0.340 | Log-linear negative binomial model |
| *C1QB* | 0.701 | Brisk | 0.044 | 0.345 | Log-linear negative binomial model |
| *CCR7* | 1.550 | Brisk | 0.044 | 0.360 | Mixture negative binomial model |
| *GZMA* | 0.872 | Brisk | 0.046 | 0.360 | Log-linear negative binomial model |
| *C1QA* | 0.632 | Brisk | 0.046 | 0.363 | Log-linear negative binomial model |
| *CRADD* | -0.920 | Non-brisk | 0.047 | 0.363 | Log-linear negative binomial model |
| *CXCL1* | 1.240 | Brisk | 0.048 | 0.363 | Log-linear negative binomial model |

| **Supplementary Table 1D.** TILs characterization of TCGA patients | | | |
| --- | --- | --- | --- |
| **Patient ID** | **TILs analysis** | **Inclusion in final analysis** | **Exclusion criteria** |
| TCGA-BF-A1PV | Brisk | Yes | - |
| TCGA-BF-A1PX | Brisk | Yes | - |
| TCGA-BF-A1Q0 | Brisk | Yes | - |
| TCGA-BF-A3DN | Brisk | Yes | - |
| TCGA-BF-A5EQ | Brisk | Yes | - |
| TCGA-BF-AAOU | Brisk | Yes | - |
| TCGA-BF-AAP7 | Brisk | Yes | - |
| TCGA-D9-A3Z4 | Brisk | Yes | - |
| TCGA-D9-A4Z3 | Brisk | Yes | - |
| TCGA-D9-A4Z5 | Brisk | Yes | - |
| TCGA-EB-A24D | Brisk | Yes | - |
| TCGA-EB-A299 | Brisk | Yes | - |
| TCGA-EB-A3XD | Brisk | Yes | - |
| TCGA-EB-A3XE | Brisk | Yes | - |
| TCGA-EB-A3XF | Brisk | Yes | - |
| TCGA-EB-A3Y6 | Brisk | Yes | - |
| TCGA-EB-A3Y7 | Brisk | Yes | - |
| TCGA-EB-A41A | Brisk | Yes | - |
| TCGA-EB-A42Y | Brisk | Yes | - |
| TCGA-EB-A42Z | Brisk | Yes | - |
| TCGA-EB-A431 | Brisk | Yes | - |
| TCGA-EB-A44N | Brisk | Yes | - |
| TCGA-EB-A44P | Brisk | Yes | - |
| TCGA-EB-A4IS | Brisk | Yes | - |
| TCGA-EB-A4XL | Brisk | Yes | - |
| TCGA-EB-A551 | Brisk | Yes | - |
| TCGA-EB-A553 | Brisk | Yes | - |
| TCGA-EB-A5UM | Brisk | Yes | - |
| TCGA-EB-A6QY | Brisk | Yes | - |
| TCGA-EB-A6QZ | Brisk | Yes | - |
| TCGA-EB-A6R0 | Brisk | Yes | - |
| TCGA-EB-A82B | Brisk | Yes | - |
| TCGA-EB-A85I | Brisk | Yes | - |
| TCGA-EB-A97M | Brisk | Yes | - |
| TCGA-ER-A2NF | Brisk | Yes | - |
| TCGA-ER-A42H | Brisk | Yes | - |
| TCGA-FR-A3R1 | Brisk | Yes | - |
| TCGA-FR-A728 | Brisk | Yes | - |
| TCGA-FS-A1ZN | Brisk | Yes | - |
| TCGA-FW-A5DX | Brisk | Yes | - |
| TCGA-GF-A769 | Brisk | Yes | - |
| TCGA-GN-A26C | Brisk | Yes | - |
| TCGA-GN-A4U5 | Brisk | Yes | - |
| TCGA-GN-A8LN | Brisk | Yes | - |
| TCGA-IH-A3EA | Brisk | Yes | - |
| TCGA-WE-A8K4 | Brisk | Yes | - |
| TCGA-XV-A9W2 | Brisk | Yes | - |
| TCGA-XV-A9W5 | Brisk | Yes | - |
| TCGA-XV-AAZV | Brisk | Yes | - |
| TCGA-XV-AAZW | Brisk | Yes | - |
| TCGA-YG-AA3N | Brisk | Yes | - |
| TCGA-BF-A1PU | Non-brisk | Yes | - |
| TCGA-BF-A1PZ | Non-brisk | Yes | - |
| TCGA-BF-A3DJ | Non-brisk | Yes | - |
| TCGA-BF-A3DM | Non-brisk | Yes | - |
| TCGA-BF-A5EO | Non-brisk | Yes | - |
| TCGA-BF-A5EP | Non-brisk | Yes | - |
| TCGA-BF-A5ES | Non-brisk | Yes | - |
| TCGA-BF-A9VF | Non-brisk | Yes | - |
| TCGA-BF-AAOX | Non-brisk | Yes | - |
| TCGA-BF-AAP1 | Non-brisk | Yes | - |
| TCGA-BF-AAP2 | Non-brisk | Yes | - |
| TCGA-BF-AAP4 | Non-brisk | Yes | - |
| TCGA-BF-AAP6 | Non-brisk | Yes | - |
| TCGA-D3-A5GT | Non-brisk | Yes | - |
| TCGA-D9-A4Z2 | Non-brisk | Yes | - |
| TCGA-EB-A1NK | Non-brisk | Yes | - |
| TCGA-EB-A24C | Non-brisk | Yes | - |
| TCGA-EB-A3HV | Non-brisk | Yes | - |
| TCGA-EB-A3XB | Non-brisk | Yes | - |
| TCGA-EB-A3XC | Non-brisk | Yes | - |
| TCGA-EB-A41B | Non-brisk | Yes | - |
| TCGA-EB-A430 | Non-brisk | Yes | - |
| TCGA-EB-A44O | Non-brisk | Yes | - |
| TCGA-EB-A4IQ | Non-brisk | Yes | - |
| TCGA-EB-A4OY | Non-brisk | Yes | - |
| TCGA-EB-A4OZ | Non-brisk | Yes | - |
| TCGA-EB-A4P0 | Non-brisk | Yes | - |
| TCGA-EB-A51B | Non-brisk | Yes | - |
| TCGA-EB-A550 | Non-brisk | Yes | - |
| TCGA-EB-A57M | Non-brisk | Yes | - |
| TCGA-EB-A5FP | Non-brisk | Yes | - |
| TCGA-EB-A5SE | Non-brisk | Yes | - |
| TCGA-EB-A5SF | Non-brisk | Yes | - |
| TCGA-EB-A5VU | Non-brisk | Yes | - |
| TCGA-EB-A82C | Non-brisk | Yes | - |
| TCGA-EB-A85J | Non-brisk | Yes | - |
| TCGA-ER-A194 | Non-brisk | Yes | - |
| TCGA-ER-A196 | Non-brisk | Yes | - |
| TCGA-ER-A19K | Non-brisk | Yes | - |
| TCGA-ER-A19T | Non-brisk | Yes | - |
| TCGA-ER-A2NB | Non-brisk | Yes | - |
| TCGA-FR-A2OS | Non-brisk | Yes | - |
| TCGA-GF-A2C7 | Non-brisk | Yes | - |
| TCGA-GN-A263 | Non-brisk | Yes | - |
| TCGA-HR-A5NC | Non-brisk | Yes | - |
| TCGA-FR-A726 | Absent | No | TILs absent |
| TCGA-BF-A3DL | Absent | No | TILs absent |
| TCGA-BF-A5ER | Limited analysis | No | Inadequate histopathological slide |
| TCGA-BF-AAP8 | Limited analysis | No | Inadequate histopathological slide |
| TCGA-DA-A960 | Limited analysis | No | Inadequate histopathological slide |
| TCGA-XV-A9VZ | Limited analysis | No | Inadequate histopathological slide |
| TCGA-XV-AAZY | Limited analysis | No | Inadequate histopathological slide |
| TCGA-GN-A269 | Non-brisk | No | RNA-seq unavaliable |

| **Supplementary Table 1E.** Differentially expressed genes in TCGA cohort | | | | |
| --- | --- | --- | --- | --- |
| **Gene** | **Log_2_ Fold Change** | **Upregulated group** | **p-value** | **Adjusted p-value** |
| *CD99* | -0.797 | Non-brisk | < 0.001 | 0.001 |
| *GBP5* | 1.353 | Brisk | < 0.001 | 0.003 |
| *PTPRC* | 1.059 | Brisk | < 0.001 | 0.005 |
| *BCAP31* | -0.636 | Non-brisk | < 0.001 | 0.006 |
| *PRKCD* | -0.592 | Non-brisk | < 0.001 | 0.006 |
| *CXCL9* | 1.630 | Brisk | < 0.001 | 0.006 |
| *SH2D1A* | 1.386 | Brisk | < 0.001 | 0.006 |
| *GPI* | -0.801 | Non-brisk | < 0.001 | 0.006 |
| *CCR5* | 0.962 | Brisk | < 0.001 | 0.006 |
| *GZMK* | 1.494 | Brisk | < 0.001 | 0.008 |
| *CXCL10* | 1.534 | Brisk | < 0.001 | 0.008 |
| *TOLLIP* | -0.597 | Non-brisk | < 0.001 | 0.008 |
| *PTPN22* | 0.799 | Brisk | < 0.001 | 0.008 |
| *IFNG* | 1.527 | Brisk | < 0.001 | 0.008 |
| *LTBR* | -0.600 | Non-brisk | < 0.001 | 0.009 |
| *STAT4* | 0.673 | Brisk | < 0.001 | 0.009 |
| *CD81* | -0.601 | Non-brisk | < 0.001 | 0.009 |
| *CD80* | 0.775 | Brisk | < 0.001 | 0.009 |
| *IRF1* | 0.796 | Brisk | < 0.001 | 0.009 |
| *CXCL11* | 1.543 | Brisk | < 0.001 | 0.009 |
| *CLEC7A* | 0.627 | Brisk | < 0.001 | 0.009 |
| *GBP1* | 1.037 | Brisk | 0.001 | 0.010 |
| *IKZF1* | 0.724 | Brisk | 0.001 | 0.010 |
| *IDO1* | 1.546 | Brisk | 0.001 | 0.010 |
| *IL15* | 0.790 | Brisk | 0.001 | 0.010 |
| *IL10RA* | 0.592 | Brisk | 0.001 | 0.010 |
| *ICAM3* | 0.920 | Brisk | 0.001 | 0.010 |
| *IL21R* | 0.918 | Brisk | 0.001 | 0.012 |
| *PSMB7* | -0.606 | Non-brisk | 0.001 | 0.012 |
| *CXCL13* | 1.603 | Brisk | 0.001 | 0.012 |
| *BTLA* | 0.982 | Brisk | 0.001 | 0.012 |
| *CD53* | 0.622 | Brisk | 0.001 | 0.012 |
| *CCR2* | 0.886 | Brisk | 0.001 | 0.012 |
| *SLAMF6* | 1.108 | Brisk | 0.001 | 0.014 |
| *IGF2R* | -0.657 | Non-brisk | 0.001 | 0.014 |
| *CDKN1A* | -0.772 | Non-brisk | 0.001 | 0.016 |
| *CD48* | 0.843 | Brisk | 0.001 | 0.016 |
| *CD163* | 0.772 | Brisk | 0.001 | 0.016 |
| *SRC* | -0.614 | Non-brisk | 0.002 | 0.016 |
| *TIGIT* | 1.024 | Brisk | 0.002 | 0.018 |
| *CD96* | 0.888 | Brisk | 0.002 | 0.018 |
| *KIT* | -1.637 | Non-brisk | 0.002 | 0.018 |
| *TAGAP* | 0.628 | Brisk | 0.002 | 0.019 |
| *IKZF3* | 1.009 | Brisk | 0.002 | 0.019 |
| *CD8B* | 1.231 | Brisk | 0.002 | 0.019 |
| *GZMA* | 0.946 | Brisk | 0.002 | 0.019 |
| *CD274* | 0.749 | Brisk | 0.002 | 0.019 |
| *PDCD1LG2* | 0.663 | Brisk | 0.002 | 0.019 |
| *MBP* | -0.710 | Non-brisk | 0.002 | 0.019 |
| *LTA* | 0.758 | Brisk | 0.003 | 0.021 |
| *ITGAL* | 0.794 | Brisk | 0.003 | 0.021 |
| *CD2* | 0.923 | Brisk | 0.003 | 0.021 |
| *LILRB1* | 0.687 | Brisk | 0.003 | 0.021 |
| *IRF8* | 0.588 | Brisk | 0.003 | 0.021 |
| *TLR7* | 0.719 | Brisk | 0.003 | 0.021 |
| *SLAMF1* | 0.828 | Brisk | 0.003 | 0.021 |
| *CD8A* | 1.139 | Brisk | 0.003 | 0.021 |
| *TNFRSF9* | 0.941 | Brisk | 0.003 | 0.022 |
| *CD247* | 0.761 | Brisk | 0.003 | 0.022 |
| *LCK* | 0.860 | Brisk | 0.003 | 0.022 |
| *HLA-DOB* | 0.798 | Brisk | 0.004 | 0.022 |
| *MSR1* | 0.664 | Brisk | 0.004 | 0.022 |
| *DUSP4* | -0.665 | Non-brisk | 0.004 | 0.023 |
| *IL2RA* | 0.616 | Brisk | 0.004 | 0.023 |
| *IRF4* | -0.810 | Non-brisk | 0.004 | 0.023 |
| *CD27* | 0.876 | Brisk | 0.004 | 0.026 |
| *CCL4* | 0.762 | Brisk | 0.005 | 0.027 |
| *CXCR6* | 0.728 | Brisk | 0.005 | 0.028 |
| *C3* | 0.926 | Brisk | 0.005 | 0.029 |
| *LAG3* | 1.006 | Brisk | 0.006 | 0.030 |
| *BST2* | 0.847 | Brisk | 0.006 | 0.030 |
| *CFB* | 0.860 | Brisk | 0.006 | 0.030 |
| *KLRD1* | 0.831 | Brisk | 0.006 | 0.030 |
| *CYBB* | 0.616 | Brisk | 0.006 | 0.030 |
| *ZAP70* | 0.734 | Brisk | 0.006 | 0.031 |
| *FADD* | -0.651 | Non-brisk | 0.006 | 0.031 |
| *NOS2* | -0.945 | Non-brisk | 0.007 | 0.033 |
| *PSMB9* | 0.646 | Brisk | 0.007 | 0.034 |
| *MS4A1* | 1.311 | Brisk | 0.008 | 0.037 |
| *IL18RAP* | 0.629 | Brisk | 0.008 | 0.037 |
| *IL7R* | 0.625 | Brisk | 0.010 | 0.044 |
| *KLRG2* | -1.096 | Non-brisk | 0.011 | 0.046 |
| *PDCD1* | 1.026 | Brisk | 0.011 | 0.046 |
| *IL2RB* | 0.632 | Brisk | 0.012 | 0.048 |
| *PYCARD* | -0.771 | Non-brisk | 0.012 | 0.048 |
| *CD7* | 0.850 | Brisk | 0.013 | 0.052 |
| *SELL* | 0.655 | Brisk | 0.014 | 0.054 |
| *CD3D* | 0.782 | Brisk | 0.014 | 0.054 |
| *TLR8* | 0.661 | Brisk | 0.014 | 0.054 |
| *CD3E* | 0.790 | Brisk | 0.014 | 0.054 |
| *CD5* | 0.642 | Brisk | 0.015 | 0.056 |
| *CXCR3* | 0.872 | Brisk | 0.015 | 0.056 |
| *LGALS3* | -0.639 | Non-brisk | 0.015 | 0.056 |
| *TBX21* | 0.698 | Brisk | 0.016 | 0.059 |
| *TNFRSF10C* | -0.788 | Non-brisk | 0.017 | 0.062 |
| *PRF1* | 0.738 | Brisk | 0.018 | 0.063 |
| *TNFRSF17* | 1.146 | Brisk | 0.020 | 0.070 |
| *CD79A* | 1.070 | Brisk | 0.023 | 0.078 |
| *CEACAM1* | -0.898 | Non-brisk | 0.024 | 0.079 |
| *CCL18* | -0.873 | Non-brisk | 0.026 | 0.085 |
| *CXCL1* | -1.032 | Non-brisk | 0.031 | 0.096 |
| *XCL1* | 0.811 | Brisk | 0.032 | 0.096 |
| *ARG1* | -1.446 | Non-brisk | 0.034 | 0.102 |
| *TLR3* | 0.604 | Brisk | 0.035 | 0.102 |
| *SLC2A1* | -0.626 | Non-brisk | 0.039 | 0.114 |
| *IL1RL2* | -0.842 | Non-brisk | 0.042 | 0.119 |
| *CEACAM6* | -1.469 | Non-brisk | 0.045 | 0.126 |
| *MASP1* | -0.775 | Non-brisk | 0.048 | 0.131 |
| *CD19* | 0.859 | Brisk | 0.048 | 0.131 |
| *HFE* | -0.754 | Non-brisk | 0.048 | 0.132 |

| **Supplementary Table 1F**. Directed global significance scores of pathways evaluated comparing TILs brisk and TILs non-brisk | | |
| --- | --- | --- |
| **Pathways** | **Directed Global Significance Scores** | **Upregulated group** |
| Cell Adhesion | 2.150 | Brisk |
| MHC Class II Antigen Presentation | 2.128 | Brisk |
| Type II Interferon Signaling | 1.988 | Brisk |
| T Cell Receptor Signaling | 1.941 | Brisk |
| Phagocytosis and Degradation | 1.917 | Brisk |
| Adaptive Immune System | 1.903 | Brisk |
| Type I Interferon Signaling | 1.834 | Brisk |
| MHC Class I Antigen Presentation | 1.797 | Brisk |
| Lymphocyte Activation | 1.704 | Brisk |
| Chemokine Signaling | 1.572 | Brisk |
| Hemostasis | 1.545 | Brisk |
| NF-kB Signaling | 1.532 | Brisk |
| Cytokine Signaling | 1.520 | Brisk |
| B cell Receptor Signaling | 1.511 | Brisk |
| Host-pathogen Interaction | 1.500 | Brisk |
| Treg Differentiation | 1.483 | Brisk |
| Lymphocyte Trafficking | 1.473 | Brisk |
| Innate Immune System | 1.439 | Brisk |
| TNF Family Signaling | 1.421 | Brisk |
| Transcriptional Regulation | 1.357 | Brisk |
| Th2 Differentiation | 1.339 | Brisk |
| Oxidative Stress | 1.295 | Brisk |
| Apoptosis | 1.253 | Brisk |
| Complement System | 1.218 | Brisk |
| TLR Signaling | 1.196 | Brisk |
| Th1 Differentiation | 1.177 | Brisk |
| NLR signaling | 1.138 | Brisk |
| Th17 Differentiation | 1.128 | Brisk |
| Autophagy | 1.085 | Brisk |
| TGF-b Signaling | 1.055 | Brisk |
| Immunometabolism | 0.915 | Brisk |
| Inflammasomes | 0.719 | Brisk |

| **Supplementary Table 1G.** Differentially expressed genes in TILs brisk cohort comparing responders and non-responders. | | | | |
| --- | --- | --- | --- | --- |
| **Genes** | **Log_2_ Fold Change** | **Upregulated group** | **p-value** | **Statistical method** |
| *DEFB103A* | 2.870 | Non-responder | 0.0458 | Simplified negative binomial model |
| *CSF2* | 2.800 | Non-responder | 0.0319 | Simplified negative binomial model |
| *KLRG2* | 2.650 | Non-responder | 0.0063 | Simplified negative binomial model |
| *ARG1* | 2.550 | Non-responder | 0.0377 | Simplified negative binomial model |
| *CCL26* | 2.360 | Non-responder | 0.0485 | Simplified negative binomial model |
| *AIRE* | 2.160 | Non-responder | 0.0144 | Simplified negative binomial model |
| *CLU* | 2.100 | Non-responder | 0.0163 | Simplified negative binomial model |
| *GATA3* | 1.910 | Non-responder | 0.00805 | Simplified negative binomial model |
| *KIR3DL2* | 1.910 | Non-responder | 0.0396 | Simplified negative binomial model |
| *CD24* | 1.780 | Non-responder | 0.0226 | Simplified negative binomial model |
| *PPARG* | 1.770 | Non-responder | 0.0285 | Simplified negative binomial model |
| *CEACAM6* | 1.700 | Non-responder | 0.049 | Simplified negative binomial model |
| *NT5E* | 1.660 | Non-responder | 0.0281 | Simplified negative binomial model |
| *IL18* | 1.490 | Non-responder | 0.0317 | Simplified negative binomial model |
| *ENTPD1* | 1.450 | Non-responder | 0.0381 | Simplified negative binomial model |
| *IL12A* | 1.280 | Non-responder | 0.00525 | Simplified negative binomial model |
| *TNFRSF4* | 1.260 | Non-responder | 0.0439 | Simplified negative binomial model |
| *LAMP3* | 1.240 | Non-responder | 0.0476 | Simplified negative binomial model |
| *PRDM1* | 1.030 | Non-responder | 0.022 | Simplified negative binomial model |
| *ZEB1* | 1.020 | Non-responder | 0.0177 | Simplified negative binomial model |
| *CARD9* | 0.933 | Non-responder | 0.0316 | Simplified negative binomial model |
| *ICOSLG* | 0.927 | Non-responder | 0.0219 | Simplified negative binomial model |
| *C5* | 0.915 | Non-responder | 0.000176 | Simplified negative binomial model |
| *IKBKE* | 0.899 | Non-responder | 0.0169 | Simplified negative binomial model |
| *BID* | 0.857 | Non-responder | 0.0432 | Simplified negative binomial model |
| *CFI* | 0.839 | Non-responder | 0.0372 | Simplified negative binomial model |
| *MYD88* | 0.792 | Non-responder | 0.0451 | Simplified negative binomial model |
| *MAPKAPK2* | 0.785 | Non-responder | 0.00281 | Simplified negative binomial model |
| *ATM* | 0.711 | Non-responder | 0.0294 | Simplified negative binomial model |
| *S1PR1* | 0.706 | Non-responder | 0.0471 | Simplified negative binomial model |
| *MAPK11* | 0.687 | Non-responder | 0.0144 | Simplified negative binomial model |
| *TNFRSF11A* | 0.605 | Non-responder | 0.0437 | Simplified negative binomial model |
| *TRAF5* | 0.588 | Non-responder | 0.016 | Simplified negative binomial model |
| *IRF4* | -0.811 | Responder | 0.0283 | Simplified negative binomial model |
| *IFIT2* | -1.190 | Responder | 0.0102 | Simplified negative binomial model |
| *MBP* | -1.590 | Responder | 0.0322 | Simplified negative binomial model |
| *SPP1* | -2.410 | Responder | 0.00697 | Simplified negative binomial model |

| **Supplementary Table 1H.** Differentially expressed genes in TILs non-brisk cohort comparing responders and non-responders. | | | | |
| --- | --- | --- | --- | --- |
| **Genes** | **Log_2_ Fold Change** | **Upregulated group** | **p-value** | **Statistical method** |
| *HLADQB1* | 3.890 | Non-responder | 0.0426 | Log-linear model (linear regression) |
| *KLRC3* | 2.950 | Non-responder | 0.0353 | Log-linear model (linear regression) |
| *KLRC4* | 2.810 | Non-responder | 0.00123 | Simplified negative binomial model |
| *CFI* | 2.260 | Non-responder | 0.00464 | Simplified negative binomial model |
| *TGFBI* | 2.190 | Non-responder | 0.00103 | Simplified negative binomial model |
| *HLADRB1* | 2.010 | Non-responder | 0.0366 | Simplified negative binomial model |
| *CXCR1* | 1.970 | Non-responder | 0.00572 | Simplified negative binomial model |
| *CLEC5A* | 1.920 | Non-responder | 0.0249 | Simplified negative binomial model |
| *TRAF1* | 1.760 | Non-responder | 0.00553 | Simplified negative binomial model |
| *IL1RAP* | 1.670 | Non-responder | 0.0135 | Simplified negative binomial model |
| *SPP1* | 1.390 | Non-responder | 0.0273 | Simplified negative binomial model |
| *C4AB* | 1.240 | Non-responder | 0.0306 | Simplified negative binomial model |
| *ZEB1* | 1.100 | Non-responder | 0.024 | Simplified negative binomial model |
| *MUC1* | 1.030 | Non-responder | 0.0442 | Simplified negative binomial model |
| *FN1* | 1.010 | Non-responder | 0.02 | Simplified negative binomial model |
| *C5* | 0.835 | Non-responder | 0.0136 | Simplified negative binomial model |
| *IL13RA1* | 0.706 | Non-responder | 0.00689 | Simplified negative binomial model |
| *IKZF2* | 0.677 | Non-responder | 0.03 | Simplified negative binomial model |
| *BCL3* | 0.640 | Non-responder | 0.0428 | Simplified negative binomial model |
| *ITGA5* | 0.636 | Non-responder | 0.0411 | Simplified negative binomial model |
| *TCF4* | 0.628 | Non-responder | 0.0346 | Simplified negative binomial model |
| *MAP4K2* | -0.653 | Responder | 0.04 | Simplified negative binomial model |
| *IKBKB* | -0.701 | Responder | 0.0248 | Simplified negative binomial model |
| *DEFB1* | -0.936 | Responder | 0.0383 | Simplified negative binomial model |
| *CFP* | -1.220 | Responder | 0.0498 | Simplified negative binomial model |
| *ITLN1* | -1.660 | Responder | 0.0271 | Simplified negative binomial model |
| *TNFSF11* | -1.760 | Responder | 0.0173 | Simplified negative binomial model |
| *IL26* | -1.860 | Responder | 0.0445 | Simplified negative binomial model |
| *PAX5* | -2.260 | Responder | 0.00912 | Simplified negative binomial model |
| *IRGM* | -2.940 | Responder | 0.00663 | Simplified negative binomial model |
| *ICAM4* | -3.430 | Responder | 0.00208 | Simplified negative binomial model |
| *NCR1* | -3.510 | Responder | 0.00135 | Simplified negative binomial model |

| **Supplementary Table 1I.** Univariate analysis and inclusion/exclusion criteria for multivariate analysis | | | | |
| --- | --- | --- | --- | --- |
| **Group** | **Variable** | **Univariate p-value** | **Inclusion in multivariable analysis** | **Exclusion reason** |
| Brisk | Breslow thicknes | 0.002294675 | Yes | - |
| Brisk | Age at diagnosis | 0.004109334 | Yes | - |
| Brisk | Clark's level | 0.054381556 | Yes | - |
| Brisk | Regression | 0.054381556 | No | Singularity/aliasing in the multivariable mode |
| Brisk | Ulceration | 0.112695481 | No | p > 0.1 |
| Brisk | Histological subtype | 0.132922543 | No | p > 0.1 |
| Brisk | Primary tumor location | 0.215074466 | No | p > 0.1 |
| Brisk | ECOG | 0.220760691 | No | p > 0.1 |
| Brisk | Neurotropism | 0.255080933 | No | p > 0.1 |
| Brisk | Line of treatment | 0.255080933 | No | p > 0.1 |
| Brisk | Stage at diagnosis | 0.29452771 | No | p > 0.1 |
| Brisk | Drug used | 0.374125358 | No | p > 0.1 |
| Brisk | Lymphovascular invasion | 0.617947191 | No | p > 0.1 |
| Brisk | Gender | 0.739779648 | No | p > 0.1 |
| Brisk | Mitotic index | 0.835378465 | No | p > 0.1 |
| Brisk | Microssatelitosis | Impossible to calculate | No | p-value not avaliable |
| Brisk | Therapeutic schema | Impossible to calculate | No | p-value not avaliable |
| Non-brisk | Regression | 0.055268698 | Yes | - |
| Non-brisk | Stage at diagnosis | 0.05937283 | Yes | - |
| Non-brisk | Line of treatment | 0.094098344 | Yes | - |
| Non-brisk | ECOG | 0.152235599 | No | p > 0.1 |
| Non-brisk | Drug used | 0.247240615 | No | p > 0.1 |
| Non-brisk | Histological subtype | 0.253631422 | No | p > 0.1 |
| Non-brisk | Age at diagnosis | 0.254592216 | No | p > 0.1 |
| Non-brisk | Neurotropism | 0.282174343 | No | p > 0.1 |
| Non-brisk | Clark's level | 0.350335039 | No | p > 0.1 |
| Non-brisk | Breslow thicknes | 0.356065095 | No | p > 0.1 |
| Non-brisk | Lymphovascular invasion | 0.527814322 | No | p > 0.1 |
| Non-brisk | Ulceration | 0.573857243 | No | p > 0.1 |
| Non-brisk | Gender | 0.604308796 | No | p > 0.1 |
| Non-brisk | Microssatelitosis | 0.715916863 | No | p > 0.1 |
| Non-brisk | Therapeutic schema | 0.715916863 | No | p > 0.1 |
| Non-brisk | Primary tumor location | 0.73651927 | No | p > 0.1 |
| Non-brisk | Mitotic index | 0.928659197 | No | p > 0.1 |

| **Supplementary Table 1J**. Directed global significance scores of pathways evaluated comparing responder and non-responder in TILs brisk cohort | | |
| --- | --- | --- |
| **Pathways** | **Directed Global Significance Scores** | **Upregulated group** |
| Complement System | 1.463 | Non-responder |
| Th1 Differentiation | 1.442 | Non-responder |
| Th2 Differentiation | 1.407 | Non-responder |
| Transcriptional Regulation | 1.330 | Non-responder |
| Immunometabolism | 1.317 | Non-responder |
| NLR signaling | 1.203 | Non-responder |
| TNF Family Signaling | 1.194 | Non-responder |
| Autophagy | 1.147 | Non-responder |
| Host-pathogen Interaction | 1.145 | Non-responder |
| Lymphocyte Activation | 1.137 | Non-responder |
| Th17 Differentiation | 1.130 | Non-responder |
| TLR Signaling | 1.119 | Non-responder |
| Innate Immune System | 1.118 | Non-responder |
| Hemostasis | 1.090 | Non-responder |
| Oxidative Stress | 1.088 | Non-responder |
| NF-kB Signaling | 1.082 | Non-responder |
| T Cell Receptor Signaling | 1.080 | Non-responder |
| Cytokine Signaling | 1.058 | Non-responder |
| Apoptosis | 0.989 | Non-responder |
| Cell Adhesion | 0.983 | Non-responder |
| MHC Class II Antigen Presentation | 0.972 | Non-responder |
| Treg Differentiation | 0.938 | Non-responder |
| Lymphocyte Trafficking | 0.931 | Non-responder |
| Chemokine Signaling | 0.925 | Non-responder |
| Adaptive Immune System | 0.914 | Non-responder |
| B cell Receptor Signaling | 0.826 | Non-responder |
| Inflammasomes | 0.805 | Non-responder |
| TGF-b Signaling | 0.804 | Non-responder |
| MHC Class I Antigen Presentation | 0.744 | Non-responder |
| Phagocytosis and Degradation | 0.730 | Non-responder |
| Type I Interferon Signaling | -0.578 | Responder |
| Type II Interferon Signaling | 0.426 | Non-responder |

| **Supplementary Table 1K**. Directed global significance scores of pathways evaluated comparing responder and non-responder in TILs non-brisk cohort | | |
| --- | --- | --- |
| **Pathways** | **Directed Global Significance Scores** | **Upregulated group** |
| Cell Adhesion | 0.859 | Non-responder |
| Autophagy | -0.830 | Responder |
| Phagocytosis and Degradation | 0.736 | Non-responder |
| MHC Class II Antigen Presentation | 0.724 | Non-responder |
| Type II Interferon Signaling | 0.706 | Non-responder |
| Complement System | 0.704 | Non-responder |
| B cell Receptor Signaling | -0.648 | Responder |
| TNF Family Signaling | 0.622 | Non-responder |
| Transcriptional Regulation | 0.621 | Non-responder |
| Th17 Differentiation | 0.610 | Non-responder |
| Lymphocyte Trafficking | 0.609 | Non-responder |
| Treg Differentiation | -0.607 | Responder |
| Adaptive Immune System | -0.542 | Responder |
| Th1 Differentiation | -0.524 | Responder |
| Host-pathogen Interaction | 0.513 | Non-responder |
| Lymphocyte Activation | 0.443 | Non-responder |
| Type I Interferon Signaling | 0.427 | Non-responder |
| Hemostasis | 0.424 | Non-responder |
| TGF-b Signaling | -0.367 | Responder |
| Th2 Differentiation | 0.353 | Non-responder |
| MHC Class I Antigen Presentation | -0.336 | Responder |
| Oxidative Stress | 0.325 | Non-responder |
| Apoptosis | 0.290 | Non-responder |
| Immunometabolism | 0.274 | Non-responder |
| TLR Signaling | -0.271 | Responder |
| Chemokine Signaling | 0.209 | Non-responder |
| NLR signaling | -0.176 | Responder |
| NF-kB Signaling | 0.153 | Non-responder |
| Inflammasomes | -0.121 | Responder |
| T Cell Receptor Signaling | 0.036 | Non-responder |

| **Supplementary Table 1L**. Pathways scores (sample level) comparing responder and non-responder in TILs brisk cohort | | | |
| --- | --- | --- | --- |
| **Pathways** | **p-value** | **Median non-responders** | **Median responders** |
| Th2 Differentiation | 0.082 | 1.097 | -0.418 |
| Transcriptional Regulation | 0.082 | 1.681 | -2.319 |
| Autophagy | 0.126 | 0.603 | -0.418 |
| B cell Receptor Signaling | 0.177 | 0.806 | -1.718 |
| Immunometabolism | 0.177 | 1.490 | -1.236 |
| Lymphocyte Activation | 0.177 | 3.672 | -6.873 |
| NF-kB Signaling | 0.177 | 1.601 | -2.416 |
| Th17 Differentiation | 0.177 | 1.235 | -0.870 |
| Complement System | 0.247 | 0.996 | -1.144 |
| TGF-b Signaling | 0.247 | 0.479 | -0.006 |
| Th1 Differentiation | 0.247 | 0.851 | -1.038 |
| Chemokine Signaling | 0.329 | 2.136 | -1.591 |
| Cytokine Signaling | 0.329 | 4.187 | -4.950 |
| NLR signaling | 0.329 | 1.699 | -1.109 |
| T Cell Receptor Signaling | 0.329 | 2.180 | -3.713 |
| TNF Family Signaling | 0.329 | 0.982 | -0.807 |
| Treg Differentiation | 0.329 | 0.525 | -0.672 |
| Hemostasis | 0.429 | 1.914 | -2.785 |
| Host-pathogen Interaction | 0.429 | 4.008 | -5.496 |
| Innate Immune System | 0.429 | 2.884 | -2.659 |
| Lymphocyte Trafficking | 0.429 | 1.176 | -1.740 |
| Oxidative Stress | 0.429 | 1.344 | -1.538 |
| Apoptosis | 0.537 | 1.811 | -1.996 |
| Inflammasomes | 0.537 | 0.515 | -0.094 |
| MHC Class II Antigen Presentation | 0.537 | 1.192 | -2.263 |
| Phagocytosis and Degradation | 0.537 | 1.430 | -1.769 |
| TLR Signaling | 0.537 | 1.845 | -2.515 |
| Adaptive Immune System | 0.792 | 2.930 | -4.677 |
| Type I Interferon Signaling | 0.792 | 0.917 | 0.542 |
| Cell Adhesion | 0.931 | 2.517 | -4.384 |
| Type II Interferon Signaling | 0.931 | 1.762 | -2.048 |
| MHC Class I Antigen Presentation | 1 | 1.413 | -1.284 |

| **Supplementary Table 1M**. Pathways scores (sample level) comparing responder and non-responder in TILs non-brisk cohort | | | |
| --- | --- | --- | --- |
| **Pathways** | **p-value** | **Median non-responders** | **Median responders** |
| Autophagy | 0.247 | -0.791 | 0.260 |
| Treg Differentiation | 0.422 | -0.584 | -0.234 |
| Transcriptional Regulation | 0.464 | 0.223 | -0.273 |
| Lymphocyte Trafficking | 0.602 | 0.185 | -0.247 |
| Type I Interferon Signaling | 0.602 | -0.705 | 0.534 |
| Adaptive Immune System | 0.651 | -1.318 | -0.095 |
| Inflammasomes | 0.651 | 0.625 | 0.139 |
| Cell Adhesion | 0.702 | -0.665 | 0.099 |
| Chemokine Signaling | 0.702 | -0.185 | -0.554 |
| Complement System | 0.702 | 0.297 | -0.398 |
| Oxidative Stress | 0.702 | 0.187 | -0.028 |
| T Cell Receptor Signaling | 0.702 | -0.666 | -0.143 |
| Type II Interferon Signaling | 0.702 | 0.209 | -0.187 |
| Lymphocyte Activation | 0.754 | -0.174 | -0.980 |
| Phagocytosis and Degradation | 0.754 | 0.804 | -1.156 |
| TLR Signaling | 0.754 | -0.494 | -0.295 |
| Apoptosis | 0.808 | -0.034 | -0.461 |
| MHC Class I Antigen Presentation | 0.808 | -0.117 | -0.330 |
| NF-kB Signaling | 0.808 | 0.288 | -0.401 |
| Th1 Differentiation | 0.808 | 0.049 | -0.333 |
| Cytokine Signaling | 0.862 | 0.688 | -0.815 |
| Host-pathogen Interaction | 0.862 | -0.483 | -0.143 |
| NLR signaling | 0.862 | -0.337 | -0.798 |
| TGF-b Signaling | 0.862 | 0.049 | 0.010 |
| Hemostasis | 0.917 | -0.639 | -0.035 |
| MHC Class II Antigen Presentation | 0.917 | 0.681 | -0.487 |
| Th17 Differentiation | 0.917 | 0.161 | -0.419 |
| Th2 Differentiation | 0.917 | -0.368 | 0.157 |
| TNF Family Signaling | 0.917 | 0.727 | -0.171 |
| Immunometabolism | 0.972 | -0.246 | 0.197 |
| Innate Immune System | 0.972 | 0.221 | -0.060 |
| B cell Receptor Signaling | 1 | 0.036 | -0.651 |

| **Supplementary Table 1N**. Cell type abundances and cell ratios comparing responder and non-responder in TILs brisk cohort | | | |
| --- | --- | --- | --- |
| **Pathways** | **p-value** | **Median non-responders** | **Median responders** |
| NK CD56^dim^ cells | 0.052 | 5.048 | 4.190 |
| NK cells | 0.126 | 5.065 | 4.398 |
| B cells | 0.126 | 6.801 | 5.254 |
| Total TILs | 0.247 | 7.828 | 6.843 |
| T cells | 0.329 | 6.902 | 5.510 |
| Th1 cells | 0.329 | 5.155 | 4.600 |
| T CD8^+^ cells | 0.329 | 6.783 | 5.213 |
| Macrophages / TILs | 0.329 | 1.587 | 2.199 |
| Neutrophils / TILs | 0.329 | 0.263 | 1.546 |
| T CD8^+^ cells/Exhasuted T CD8^+^ cells | 0.329 | 0.474 | -0.125 |
| Cytotoxic cells | 0.429 | 7.189 | 6.139 |
| T_reg_ cells | 0.429 | 6.295 | 5.787 |
| T cells / TILs | 0.429 | -0.821 | -1.101 |
| T CD8^+^ cells / T cells | 0.429 | -0.079 | 0.225 |
| CD45 ^+^ cells | 0.537 | 8.681 | 7.721 |
| Dendritic cells | 0.537 | 7.357 | 7.042 |
| T_reg_ cells / TILs | 0.537 | -1.594 | -0.854 |
| T CD8^+^ cells / T_reg_ cells | 0.537 | 0.627 | -0.329 |
| NK CD56^dim^ cells / TILs | 0.662 | -2.773 | -2.892 |
| Th1 cells / TILs | 0.662 | -2.386 | -2.259 |
| Exhausted T CD8^+^ cells | 0.792 | 6.470 | 5.767 |
| Macrophages | 0.792 | 9.243 | 9.000 |
| Cytotoxic cells / TILs | 0.792 | -0.532 | -0.704 |
| Exhausted T CD8^+^ cells / TILs | 0.792 | -1.254 | -1.465 |
| T CD8^+^ cells / TILs | 0.792 | -1.047 | -0.630 |
| Neutrophils | 0.931 | 8.193 | 8.389 |
| NK cells / TILs | 0.931 | -2.888 | -2.845 |
| B cells / TILs | 1.000 | -1.050 | -1.324 |
| Dendritic cells / TILs | 1.000 | -0.532 | 0.323 |

| **Supplementary Table 1O**. Cell type abundances and cell ratios comparing responder and non-responder in TILs non-brisk cohort | | | |
| --- | --- | --- | --- |
| **Pathways** | **p-value** | **Median non-responders** | **Median responders** |
| Neutrophils | 0.111 | 7.175 | 6.889 |
| Macrophages / TILs | 0.148 | 2.595 | 2.302 |
| Macrophages | 0.169 | 8.924 | 8.178 |
| Cytotoxic cells / TILs | 0.219 | -0.698 | -0.455 |
| Neutrophils / TILs | 0.247 | 0.951 | 0.723 |
| NK CD56dim cells / TILs | 0.277 | -2.147 | -1.835 |
| T cells / TILs | 0.277 | -1.347 | -1.100 |
| NK CD56dim cells | 0.310 | 3.460 | 4.060 |
| Exhausted T CD8+ cells / TILs | 0.382 | -0.809 | -0.544 |
| Treg cells / TILs | 0.422 | -1.683 | -1.558 |
| Th1 cells / TILs | 0.464 | -2.204 | -2.066 |
| T CD8+ cells / TILs | 0.464 | -1.258 | -1.158 |
| Exhausted T CD8+ cells | 0.508 | 5.192 | 5.227 |
| Cytotoxic cells | 0.602 | 5.239 | 5.590 |
| T cells | 0.651 | 4.488 | 4.982 |
| Dendritic cells | 0.651 | 6.159 | 6.144 |
| Th1 cells | 0.702 | 3.682 | 3.909 |
| T CD8+ cells | 0.702 | 4.447 | 4.807 |
| Treg cells | 0.702 | 4.384 | 4.618 |
| T CD8+ cells / T cells | 0.808 | -0.043 | -0.131 |
| B cells / TILs | 0.862 | -1.510 | -1.326 |
| T CD8+ cells / Treg cells | 0.862 | 0.553 | 0.435 |
| CD45 + cells | 0.917 | 6.750 | 6.714 |
| Dendritic cells / TILs | 0.917 | -0.008 | -0.024 |
| B cells | 0.972 | 4.714 | 4.502 |
| Total TILs | 0.972 | 6.089 | 5.989 |
| NK cells | 0.972 | 3.785 | 3.756 |
| NK cells / TILs | 0.972 | -2.170 | -2.169 |
| T CD8+ cells/Exhasuted T CD8+ cells | 1.000 | -0.481 | -0.598 |

| **Supplementary Table 1P**. Association of gene expression and survival endpoints in TILs brisk cohort | | | | | | |
| --- | --- | --- | --- | --- | --- | --- |
| **Gene** | **Survival endpoint** | **HR** | **HR Lower 95% CI** | **HR Upper 95% CI** | **p-value** | **Adjusted p-value** |
| *TNFRSF4* | Overall survival | 3.708 | 1.238 | 11.106 | 0.019 | 0.218 |
| *TRAF5* | Overall survival | 2.302 | 1.113 | 4.759 | 0.025 | 0.218 |
| *KIR3DL2* | Overall survival | 3.132 | 1.149 | 8.538 | 0.026 | 0.218 |
| *IKBKE* | Overall survival | 2.500 | 1.079 | 5.793 | 0.033 | 0.218 |
| *MAPKAPK2* | Overall survival | 2.920 | 1.080 | 7.898 | 0.035 | 0.218 |
| *KLRG2* | Overall survival | 2.845 | 1.070 | 7.566 | 0.036 | 0.218 |
| *CARD9* | Overall survival | 2.519 | 1.038 | 6.114 | 0.041 | 0.218 |
| *PPARG* | Overall survival | 2.283 | 1.032 | 5.046 | 0.041 | 0.218 |
| *S1PR1* | Overall survival | 2.518 | 0.969 | 6.544 | 0.058 | 0.223 |
| *C5* | Overall survival | 1.888 | 0.945 | 3.773 | 0.072 | 0.223 |
| *SPP1* | Overall survival | 0.241 | 0.051 | 1.142 | 0.073 | 0.223 |
| *NT5E* | Overall survival | 2.163 | 0.927 | 5.046 | 0.074 | 0.223 |
| *AIRE* | Overall survival | 1.962 | 0.901 | 4.271 | 0.089 | 0.235 |
| *MAPK11* | Overall survival | 1.840 | 0.887 | 3.817 | 0.101 | 0.235 |
| *MYD88* | Overall survival | 1.982 | 0.864 | 4.548 | 0.106 | 0.235 |
| *ICOSLG* | Overall survival | 1.682 | 0.876 | 3.226 | 0.118 | 0.237 |
| *IFIT2* | Overall survival | 0.430 | 0.147 | 1.261 | 0.124 | 0.237 |
| *DEFB103A* | Overall survival | 4.457 | 0.599 | 33.191 | 0.145 | 0.251 |
| *IL12A* | Overall survival | 1.824 | 0.806 | 4.130 | 0.149 | 0.251 |
| *IRF4* | Overall survival | 0.371 | 0.090 | 1.527 | 0.169 | 0.274 |
| *IL18* | Overall survival | 1.522 | 0.813 | 2.848 | 0.189 | 0.295 |
| *CD24* | Overall survival | 1.504 | 0.777 | 2.912 | 0.226 | 0.312 |
| *ZEB1* | Overall survival | 1.531 | 0.767 | 3.058 | 0.227 | 0.312 |
| *ENTPD1* | Overall survival | 1.530 | 0.766 | 3.059 | 0.229 | 0.312 |
| *ATM* | Overall survival | 1.510 | 0.770 | 2.958 | 0.230 | 0.312 |
| *CFI* | Overall survival | 1.678 | 0.708 | 3.977 | 0.240 | 0.314 |
| *TNFRSF11A* | Overall survival | 1.536 | 0.743 | 3.178 | 0.247 | 0.314 |
| *PRDM1* | Overall survival | 1.414 | 0.775 | 2.582 | 0.259 | 0.320 |
| *GATA3* | Overall survival | 1.389 | 0.754 | 2.557 | 0.292 | 0.341 |
| *CLU* | Overall survival | 1.430 | 0.712 | 2.873 | 0.315 | 0.357 |
| *BID* | Overall survival | 1.461 | 0.665 | 3.211 | 0.345 | 0.381 |
| *ARG1* | Overall survival | 1.301 | 0.722 | 2.345 | 0.381 | 0.410 |
| *CSF2* | Overall survival | 1.408 | 0.641 | 3.095 | 0.394 | 0.414 |
| *MBP* | Overall survival | 0.986 | 0.502 | 1.937 | 0.968 | 0.968 |
| *TRAF5* | Progression-free survival | 34.585 | 1.014 | 1179.311 | 0.049 | 0.233 |
| *MYD88* | Progression-free survival | 4.561 | 0.983 | 21.159 | 0.053 | 0.601 |
| *IL12A* | Progression-free survival | 3.142 | 0.971 | 10.171 | 0.056 | 0.440 |
| *S1PR1* | Progression-free survival | 3.001 | 0.971 | 9.276 | 0.056 | 0.862 |
| *KIR3DL2* | Progression-free survival | 4.131 | 0.962 | 17.748 | 0.056 | 0.664 |
| *ENTPD1* | Progression-free survival | 4.936 | 0.956 | 25.489 | 0.057 | 0.502 |
| *MAPK11* | Progression-free survival | 2.679 | 0.952 | 7.541 | 0.062 | 0.938 |
| *PPARG* | Progression-free survival | 2.488 | 0.921 | 6.721 | 0.072 | 0.818 |
| *MAPKAPK2* | Progression-free survival | 3.777 | 0.870 | 16.391 | 0.076 | 0.962 |
| *TNFRSF4* | Progression-free survival | 2.268 | 0.882 | 5.834 | 0.089 | 0.033 |
| *IKBKE* | Progression-free survival | 2.438 | 0.847 | 7.013 | 0.098 | 0.760 |
| *IRF4* | Progression-free survival | 0.045 | 0.001 | 1.955 | 0.107 | 0.958 |
| *KLRG2* | Progression-free survival | 1.974 | 0.836 | 4.662 | 0.121 | 0.084 |
| *CFI* | Progression-free survival | 2.533 | 0.763 | 8.411 | 0.129 | 0.951 |
| *C5* | Progression-free survival | 1.840 | 0.802 | 4.221 | 0.150 | 0.549 |
| *ATM* | Progression-free survival | 1.786 | 0.798 | 3.996 | 0.158 | 0.827 |
| *AIRE* | Progression-free survival | 1.934 | 0.768 | 4.869 | 0.162 | 0.965 |
| *ZEB1* | Progression-free survival | 1.722 | 0.776 | 3.819 | 0.181 | 0.893 |
| *BID* | Progression-free survival | 1.824 | 0.748 | 4.449 | 0.186 | 0.754 |
| *CARD9* | Progression-free survival | 1.796 | 0.722 | 4.469 | 0.208 | 0.533 |
| *ICOSLG* | Progression-free survival | 1.627 | 0.748 | 3.541 | 0.220 | 0.484 |
| *SPP1* | Progression-free survival | 0.160 | 0.008 | 3.089 | 0.225 | 0.393 |
| *DEFB103A* | Progression-free survival | 1.820 | 0.675 | 4.903 | 0.236 | 0.592 |
| *CD24* | Progression-free survival | 1.562 | 0.707 | 3.453 | 0.270 | 0.944 |
| *NT5E* | Progression-free survival | 1.586 | 0.695 | 3.621 | 0.273 | 0.110 |
| *CLU* | Progression-free survival | 1.541 | 0.676 | 3.514 | 0.303 | 0.259 |
| *IL18* | Progression-free survival | 1.479 | 0.701 | 3.123 | 0.304 | 0.524 |
| *PRDM1* | Progression-free survival | 1.432 | 0.714 | 2.873 | 0.311 | 0.232 |
| *IFIT2* | Progression-free survival | 0.617 | 0.238 | 1.599 | 0.320 | 0.277 |
| *GATA3* | Progression-free survival | 1.425 | 0.699 | 2.905 | 0.330 | 0.773 |
| *TNFRSF11A* | Progression-free survival | 1.463 | 0.632 | 3.387 | 0.374 | 0.263 |
| *MBP* | Progression-free survival | 0.741 | 0.327 | 1.676 | 0.472 | 0.351 |
| *ARG1* | Progression-free survival | 1.246 | 0.614 | 2.528 | 0.542 | 0.292 |
| *CSF2* | Progression-free survival | 1.098 | 0.448 | 2.693 | 0.838 | 0.287 |
| *TNFRSF4* | Post-immunotherapy survival | 3.593 | 1.216 | 10.620 | 0.021 | 0.325 |
| *KIR3DL2* | Post-immunotherapy survival | 2.862 | 1.107 | 7.398 | 0.030 | 0.377 |
| *KLRG2* | Post-immunotherapy survival | 2.802 | 1.063 | 7.383 | 0.037 | 0.514 |
| *CARD9* | Post-immunotherapy survival | 2.560 | 1.047 | 6.261 | 0.039 | 0.460 |
| *IKBKE* | Post-immunotherapy survival | 2.269 | 1.005 | 5.124 | 0.049 | 0.063 |
| *MAPKAPK2* | Post-immunotherapy survival | 2.409 | 1.003 | 5.784 | 0.049 | 0.441 |
| *TRAF5* | Post-immunotherapy survival | 1.889 | 0.991 | 3.598 | 0.053 | 0.188 |
| *PPARG* | Post-immunotherapy survival | 2.120 | 0.958 | 4.690 | 0.064 | 0.289 |
| *S1PR1* | Post-immunotherapy survival | 2.425 | 0.928 | 6.340 | 0.071 | 0.964 |
| *NT5E* | Post-immunotherapy survival | 2.166 | 0.928 | 5.056 | 0.074 | 0.455 |
| *C5* | Post-immunotherapy survival | 1.862 | 0.928 | 3.736 | 0.080 | 0.217 |
| *SPP1* | Post-immunotherapy survival | 0.241 | 0.048 | 1.225 | 0.086 | 0.898 |
| *AIRE* | Post-immunotherapy survival | 1.978 | 0.893 | 4.382 | 0.093 | 0.694 |
| *IFIT2* | Post-immunotherapy survival | 0.452 | 0.165 | 1.235 | 0.122 | 0.644 |
| *ICOSLG* | Post-immunotherapy survival | 1.636 | 0.848 | 3.157 | 0.142 | 0.105 |
| *MYD88* | Post-immunotherapy survival | 1.753 | 0.811 | 3.791 | 0.154 | 0.343 |
| *MAPK11* | Post-immunotherapy survival | 1.675 | 0.824 | 3.407 | 0.154 | 0.383 |
| *IL12A* | Post-immunotherapy survival | 1.666 | 0.766 | 3.624 | 0.198 | 0.414 |
| *IRF4* | Post-immunotherapy survival | 0.403 | 0.096 | 1.697 | 0.215 | 0.711 |
| *DEFB103A* | Post-immunotherapy survival | 6.407 | 0.333 | 123.406 | 0.218 | 0.751 |
| *TNFRSF11A* | Post-immunotherapy survival | 1.568 | 0.760 | 3.233 | 0.224 | 0.581 |
| *IL18* | Post-immunotherapy survival | 1.472 | 0.780 | 2.777 | 0.233 | 0.062 |
| *ZEB1* | Post-immunotherapy survival | 1.509 | 0.757 | 3.009 | 0.242 | 0.450 |
| *CFI* | Post-immunotherapy survival | 1.629 | 0.707 | 3.754 | 0.252 | 0.683 |
| *CD24* | Post-immunotherapy survival | 1.461 | 0.752 | 2.840 | 0.264 | 0.202 |
| *PRDM1* | Post-immunotherapy survival | 1.395 | 0.761 | 2.558 | 0.281 | 0.203 |
| *ATM* | Post-immunotherapy survival | 1.443 | 0.732 | 2.842 | 0.290 | 0.352 |
| *CLU* | Post-immunotherapy survival | 1.443 | 0.711 | 2.931 | 0.310 | 0.582 |
| *GATA3* | Post-immunotherapy survival | 1.362 | 0.733 | 2.530 | 0.328 | 0.245 |
| *CSF2* | Post-immunotherapy survival | 1.454 | 0.674 | 3.135 | 0.339 | 0.412 |
| *ENTPD1* | Post-immunotherapy survival | 1.358 | 0.703 | 2.624 | 0.363 | 0.169 |
| *BID* | Post-immunotherapy survival | 1.408 | 0.649 | 3.054 | 0.386 | 0.940 |
| *ARG1* | Post-immunotherapy survival | 1.296 | 0.718 | 2.340 | 0.389 | 0.268 |
| *MBP* | Post-immunotherapy survival | 0.989 | 0.505 | 1.939 | 0.974 | 0.185 |
| HR: Hazard ratio. CI: Confidence interval. | | | | | | |

| **Supplementary Table 1Q** Association of gene expression and survival endpoints in TILs non-brisk cohort | | | | | | |
| --- | --- | --- | --- | --- | --- | --- |
| **Gene** | **Survival endpoint** | **HR** | **HR Lower 95% CI** | **HR Upper 95% CI** | **p-value** | **Adjusted p-value** |
| *HLA-DRB1* | Overall survival | 4.440 | 1.874 | 10.524 | 0.001 | 0.637 |
| *CLEC5A* | Overall survival | 2.872 | 1.333 | 6.189 | 0.007 | 0.070 |
| *FN1* | Overall survival | 1.965 | 1.169 | 3.303 | 0.011 | 0.801 |
| *HLA-DQB1* | Overall survival | 1.499 | 1.005 | 2.236 | 0.047 | 0.486 |
| *MAP4K2* | Overall survival | 0.526 | 0.250 | 1.107 | 0.091 | 0.858 |
| *TRAF1* | Overall survival | 1.506 | 0.924 | 2.457 | 0.101 | 0.727 |
| *ICAM4* | Overall survival | 0.242 | 0.040 | 1.456 | 0.121 | 0.893 |
| *IRGM* | Overall survival | 0.632 | 0.304 | 1.312 | 0.218 | 0.103 |
| *IKBKB* | Overall survival | 0.725 | 0.416 | 1.263 | 0.256 | 0.025 |
| *IKZF2* | Overall survival | 1.260 | 0.833 | 1.906 | 0.273 | 0.267 |
| *IL13RA1* | Overall survival | 1.234 | 0.836 | 1.821 | 0.289 | 0.262 |
| *CFI* | Overall survival | 1.288 | 0.780 | 2.127 | 0.323 | 0.478 |
| *CXCR1* | Overall survival | 1.206 | 0.814 | 1.787 | 0.350 | 0.649 |
| *KLRC4* | Overall survival | 1.212 | 0.769 | 1.909 | 0.408 | 0.049 |
| *SPP1* | Overall survival | 1.245 | 0.729 | 2.126 | 0.422 | 0.962 |
| *PAX5* | Overall survival | 0.795 | 0.445 | 1.419 | 0.437 | 0.358 |
| *KLRC3* | Overall survival | 1.198 | 0.755 | 1.900 | 0.444 | 0.127 |
| *MUC1* | Overall survival | 1.202 | 0.730 | 1.980 | 0.470 | 0.105 |
| *IL1RAP* | Overall survival | 1.145 | 0.782 | 1.678 | 0.486 | 0.132 |
| *ZEB1* | Overall survival | 1.110 | 0.730 | 1.688 | 0.627 | 0.264 |
| *C5* | Overall survival | 1.111 | 0.721 | 1.712 | 0.634 | 0.037 |
| *TCF4* | Overall survival | 0.897 | 0.544 | 1.478 | 0.670 | 0.064 |
| *CFP* | Overall survival | 0.899 | 0.536 | 1.507 | 0.686 | 0.009 |
| *C4A/B* | Overall survival | 1.114 | 0.660 | 1.881 | 0.686 | 0.228 |
| *ITGA5* | Overall survival | 1.089 | 0.651 | 1.822 | 0.744 | 0.785 |
| *IL26* | Overall survival | 1.049 | 0.649 | 1.697 | 0.845 | 0.006 |
| *NCR1* | Overall survival | 1.047 | 0.625 | 1.753 | 0.861 | 0.021 |
| *ITLN1* | Overall survival | 0.952 | 0.540 | 1.677 | 0.864 | 0.013 |
| *BCL3* | Overall survival | 1.031 | 0.648 | 1.640 | 0.898 | 0.182 |
| *TGFBI* | Overall survival | 1.026 | 0.661 | 1.594 | 0.907 | 0.167 |
| *DEFB1* | Overall survival | 1.020 | 0.653 | 1.593 | 0.930 | 0.035 |
| *TNFSF11* | Overall survival | 1.008 | 0.649 | 1.565 | 0.973 | 0.078 |
| *ZEB1* | Progression-free survival | 2.655 | 1.296 | 5.441 | 0.008 | 0.797 |
| *CFP* | Progression-free survival | 0.406 | 0.176 | 0.935 | 0.034 | 0.615 |
| *FN1* | Progression-free survival | 1.819 | 1.039 | 3.184 | 0.036 | 0.419 |
| *CLEC5A* | Progression-free survival | 1.722 | 0.950 | 3.122 | 0.073 | 0.620 |
| *IKBKB* | Progression-free survival | 0.576 | 0.311 | 1.069 | 0.080 | 0.461 |
| *KLRC3* | Progression-free survival | 1.637 | 0.915 | 2.928 | 0.097 | 0.371 |
| *DEFB1* | Progression-free survival | 0.454 | 0.168 | 1.224 | 0.118 | 0.491 |
| *CFI* | Progression-free survival | 1.447 | 0.903 | 2.317 | 0.124 | 0.264 |
| *IRGM* | Progression-free survival | 0.586 | 0.293 | 1.172 | 0.131 | 0.920 |
| *TNFSF11* | Progression-free survival | 0.502 | 0.199 | 1.265 | 0.144 | 0.642 |
| *C4A/B* | Progression-free survival | 1.557 | 0.852 | 2.843 | 0.150 | 0.851 |
| *SPP1* | Progression-free survival | 1.477 | 0.866 | 2.518 | 0.152 | 0.106 |
| *IL26* | Progression-free survival | 0.682 | 0.400 | 1.164 | 0.161 | 0.480 |
| *HLA-DRB1* | Progression-free survival | 1.483 | 0.846 | 2.598 | 0.168 | 0.893 |
| *TCF4* | Progression-free survival | 1.490 | 0.815 | 2.726 | 0.196 | 0.605 |
| *ITLN1* | Progression-free survival | 0.646 | 0.330 | 1.263 | 0.201 | 0.413 |
| *TRAF1* | Progression-free survival | 1.438 | 0.815 | 2.539 | 0.210 | 0.979 |
| *MAP4K2* | Progression-free survival | 0.692 | 0.389 | 1.231 | 0.210 | 0.382 |
| *IL13RA1* | Progression-free survival | 1.504 | 0.780 | 2.901 | 0.223 | 0.110 |
| *KLRC4* | Progression-free survival | 1.467 | 0.783 | 2.752 | 0.232 | 0.284 |
| *CXCR1* | Progression-free survival | 0.738 | 0.434 | 1.254 | 0.261 | 0.585 |
| *PAX5* | Progression-free survival | 0.746 | 0.417 | 1.335 | 0.324 | 0.145 |
| *IKZF2* | Progression-free survival | 0.782 | 0.471 | 1.298 | 0.341 | 0.145 |
| *NCR1* | Progression-free survival | 0.823 | 0.438 | 1.547 | 0.545 | 0.541 |
| *IL1RAP* | Progression-free survival | 1.136 | 0.690 | 1.869 | 0.617 | 0.155 |
| *HLA-DQB1* | Progression-free survival | 1.127 | 0.700 | 1.816 | 0.622 | 0.615 |
| *ITGA5* | Progression-free survival | 1.135 | 0.677 | 1.902 | 0.631 | 0.290 |
| *MUC1* | Progression-free survival | 0.899 | 0.536 | 1.508 | 0.687 | 0.230 |
| *BCL3* | Progression-free survival | 0.904 | 0.530 | 1.541 | 0.710 | 0.068 |
| *TGFBI* | Progression-free survival | 1.081 | 0.648 | 1.802 | 0.766 | 0.312 |
| *C5* | Progression-free survival | 0.945 | 0.539 | 1.657 | 0.844 | 0.986 |
| *ICAM4* | Progression-free survival | 0.993 | 0.635 | 1.551 | 0.975 | 0.273 |
| *HLA-DRB1* | Post-immunotherapy survival | 2.699 | 1.400 | 5.204 | 0.003 | 0.109 |
| *CLEC5A* | Post-immunotherapy survival | 2.143 | 1.164 | 3.943 | 0.014 | 0.257 |
| *FN1* | Post-immunotherapy survival | 1.693 | 1.081 | 2.650 | 0.021 | 0.257 |
| *MAP4K2* | Post-immunotherapy survival | 0.470 | 0.223 | 0.987 | 0.046 | 0.324 |
| *IL13RA1* | Post-immunotherapy survival | 1.553 | 0.993 | 2.429 | 0.054 | 0.324 |
| *HLA-DQB1* | Post-immunotherapy survival | 1.465 | 0.959 | 2.238 | 0.077 | 0.397 |
| *ICAM4* | Post-immunotherapy survival | 0.323 | 0.081 | 1.282 | 0.108 | 0.486 |
| *TRAF1* | Post-immunotherapy survival | 1.475 | 0.894 | 2.435 | 0.128 | 0.514 |
| *IRGM* | Post-immunotherapy survival | 0.679 | 0.358 | 1.288 | 0.237 | 0.697 |
| *ZEB1* | Post-immunotherapy survival | 1.300 | 0.815 | 2.074 | 0.271 | 0.697 |
| *MUC1* | Post-immunotherapy survival | 1.295 | 0.794 | 2.114 | 0.300 | 0.697 |
| *IKBKB* | Post-immunotherapy survival | 0.758 | 0.447 | 1.283 | 0.302 | 0.697 |
| *IKZF2* | Post-immunotherapy survival | 1.205 | 0.821 | 1.769 | 0.340 | 0.697 |
| *IL1RAP* | Post-immunotherapy survival | 1.204 | 0.808 | 1.793 | 0.362 | 0.697 |
| *C5* | Post-immunotherapy survival | 1.220 | 0.795 | 1.873 | 0.363 | 0.697 |
| *CFI* | Post-immunotherapy survival | 1.260 | 0.762 | 2.086 | 0.368 | 0.697 |
| *SPP1* | Post-immunotherapy survival | 1.263 | 0.737 | 2.165 | 0.395 | 0.712 |
| *NCR1* | Post-immunotherapy survival | 1.208 | 0.732 | 1.994 | 0.459 | 0.787 |
| *BCL3* | Post-immunotherapy survival | 1.164 | 0.734 | 1.847 | 0.519 | 0.800 |
| *PAX5* | Post-immunotherapy survival | 0.832 | 0.471 | 1.470 | 0.527 | 0.800 |
| *CXCR1* | Post-immunotherapy survival | 1.133 | 0.765 | 1.679 | 0.533 | 0.800 |
| *ITGA5* | Post-immunotherapy survival | 1.151 | 0.678 | 1.952 | 0.603 | 0.868 |
| *TGFBI* | Post-immunotherapy survival | 1.087 | 0.695 | 1.699 | 0.716 | 0.953 |
| *TCF4* | Post-immunotherapy survival | 1.091 | 0.649 | 1.833 | 0.744 | 0.953 |
| *CFP* | Post-immunotherapy survival | 0.935 | 0.588 | 1.488 | 0.777 | 0.953 |
| *ITLN1* | Post-immunotherapy survival | 1.076 | 0.619 | 1.871 | 0.794 | 0.953 |
| *C4A/B* | Post-immunotherapy survival | 0.956 | 0.578 | 1.580 | 0.860 | 0.959 |
| *IL26* | Post-immunotherapy survival | 1.036 | 0.667 | 1.607 | 0.875 | 0.959 |
| *KLRC4* | Post-immunotherapy survival | 1.031 | 0.666 | 1.594 | 0.892 | 0.959 |
| *KLRC3* | Post-immunotherapy survival | 1.020 | 0.654 | 1.591 | 0.930 | 0.959 |
| *TNFSF11* | Post-immunotherapy survival | 0.982 | 0.654 | 1.477 | 0.932 | 0.959 |
| *DEFB1* | Post-immunotherapy survival | 1.007 | 0.674 | 1.506 | 0.971 | 0.971 |
| HR: Hazard ratio. CI: Confidence interval. | | | | | | |
